# GDF6-CD99 Signaling Regulates Src and Ewing Sarcoma Growth

**DOI:** 10.1016/j.celrep.2020.108332

**Published:** 2020-11-03

**Authors:** Fuchun Zhou, David J. Elzi, Panneerselvam Jayabal, Xiuye Ma, Yu-Chiao Chiu, Yidong Chen, Barron Blackman, Susan T. Weintraub, Peter J. Houghton, Yuzuru Shiio

**Affiliations:** 1 Greehey Children’s Cancer Research Institute, The University of Texas Health Science Center, San Antonio, TX 78229, USA; 2 BioAffinity Technologies, Inc., 1 UTSA Circle, San Antonio, TX 78249, USA; 3 Department of Population Health Sciences, The University of Texas Health Science Center, San Antonio, TX 78229, USA; 4 Mays Cancer Center, The University of Texas Health Science Center, San Antonio, TX 78229, USA; 5 Department of Biochemistry and Structural Biology, The University of Texas Health Science Center, San Antonio, TX 78229, USA; 6 Department of Molecular Medicine, The University of Texas Health Science Center, San Antonio, TX 78229, USA; 7 Lead Contact

## Abstract

We report here that the autocrine signaling mediated by growth and differentiation factor 6 (GDF6), a member of the bone morphogenetic protein (BMP) family of cytokines, maintains Ewing sarcoma growth by preventing Src hyperactivation. Surprisingly, Ewing sarcoma depends on the prodomain, not the BMP domain, of GDF6. We demonstrate that the GDF6 prodomain is a ligand for CD99, a transmembrane protein that has been widely used as a marker of Ewing sarcoma. The binding of the GDF6 prodomain to the CD99 extracellular domain results in recruitment of CSK (C-terminal Src kinase) to the YQKKK motif in the intracellular domain of CD99, inhibiting Src activity. GDF6 silencing causes hyperactivation of Src and p21-dependent growth arrest. We demonstrate that two GDF6 prodomain mutants linked to Klippel-Feil syndrome are hyperactive in CD99-Src signaling. These results reveal a cytokine signaling pathway that regulates the CSK-Src axis and cancer cell proliferation and suggest the gain-of-function activity for disease-causing GDF6 mutants.

## In Brief

Ewing sarcoma is driven by the EWS-ETS fusion oncoprotein, but little is known about the extracellular signaling regulating this cancer. Zhou et al. report that the prodomain of GDF6 is a ligand for CD99, inhibiting Src through CSK and maintaining Ewing sarcoma growth in an autocrine fashion.

## Graphical Abstract

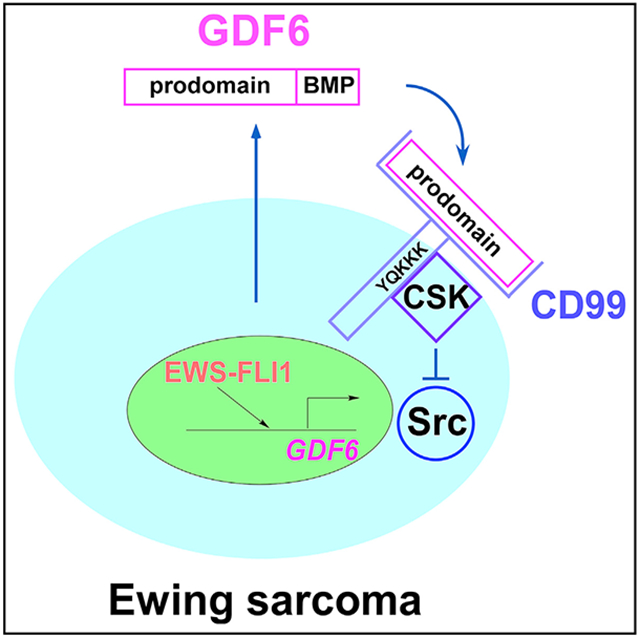

## INTRODUCTION

Ewing sarcoma is a cancer of bone and soft tissue in children that is characterized by a chromosomal translocation generating a fusion between EWS and an Ets family transcription factor, most commonly FLI1 (Jedlicka, 2010; Lawlor and Sorensen, 2015; Lessnick and Ladanyi, 2012; Mackintosh et al., 2010; Toomey et al., 2010). EWS-FLI1 fusion accounts for 85% of the cases. EWS-FLI1 regulates the expression of a number of genes important for cell proliferation and tumor progression (Hancock and Lessnick, 2008), can transform mouse cells (González et al., 2007; May et al., 1993), and is required for proliferation and tumorigenicity of Ewing sarcoma cells. Therefore, EWS-FLI1 is considered a driver for Ewing sarcoma. Previous research on Ewing sarcoma largely focused on intracellular pathways. Little is known about the extracellular signaling that regulates Ewing sarcoma.

A transmembrane protein, CD99, is highly expressed in Ewing sarcoma and has been commonly used as a marker for this cancer (Ambros et al., 1991; Fellinger et al., 1991; Kovar et al., 1990). The silencing of CD99 was shown to inhibit the growth and migration of Ewing sarcoma cells (Kreppel et al., 2006) or to induce neural differentiation in Ewing sarcoma cells and reduce their tumorigenicity and bone metastasis capability (Rocchi et al., 2010). EWS-FLI1 induces the expression of CD99, either by direct binding to the *CD99* gene promoter (Rocchi et al., 2010) or through regulation of a microRNA (miRNA) (Franzetti et al., 2013). Targeting CD99 by antibody-mediated engagement caused caspase-independent cell death, methuosis, in Ewing sarcoma (Cerisano et al., 2004; Manara et al., 2016; Scotlandi et al., 2000). Recently, clofarabine was identified as a compound that binds CD99 and inhibits Ewing sarcoma growth (Çelik et al., 2018). These studies suggested the importance of CD99 in Ewing sarcoma biology and the need to understand its mechanism of action (Pasello et al., 2018).

Growth and differentiation factor 6 (GDF6; also known as bone morphogenetic protein 13 [BMP13]) is a cytokine of the BMP family. It is highly conserved in vertebrates and has been implicated in skeletal and eye development (Williams et al., 2008). GDF6 is synthesized as a precursor that has the prodomain at the N terminus and the BMP domain at the C terminus; a proteolytic cleavage in Golgi separates the two domains. The BMP domain of GDF6 binds to BMP receptors and stimulates Smad1, Smad5, and Smad8. Mutations in the prodomain of GDF6 are associated with several developmental abnormalities of skeleton, eye, and other organs, including Klippel-Feil syndrome (Williams et al., 2008). Despite the link to developmental abnormalities, the function of the GDF6 prodomain has not been studied.

In this study, we describe a GDF6 prodomain signaling pathway that regulates Src activity and Ewing sarcoma growth. Our results indicate that GDF6 prodomain uses CD99 as the receptor, recruiting CSK (C-terminal Src kinase) to the CD99 intracellular domain and inhibiting Src. GDF6 prodomain mutants linked to Klippel-Feil syndrome are hyperactive in GDF6-CD99-Src signaling.

## RESULTS

### Secretome Proteomics Identifies GDF6 as a Target of EWS-FLI1

To dissect the cytokine signaling in Ewing sarcoma, we analyzed the proteins secreted from A673 and TC71 Ewing sarcoma cells by mass spectrometry. The complete lists of proteins identified with high confidence are shown in Table S1. This analysis identified the secretion of GDF6 from both Ewing sarcoma cell lines (Table S1). GDF6 secretion was not detected in the secretome of other childhood sarcomas, including rhabdomyosarcoma (RH30 and RD; Table S1), osteosarcoma (U2OS and Saos-2; Table S1), and synovial sarcoma (Yamato; Table S1).

**Figure 1.**
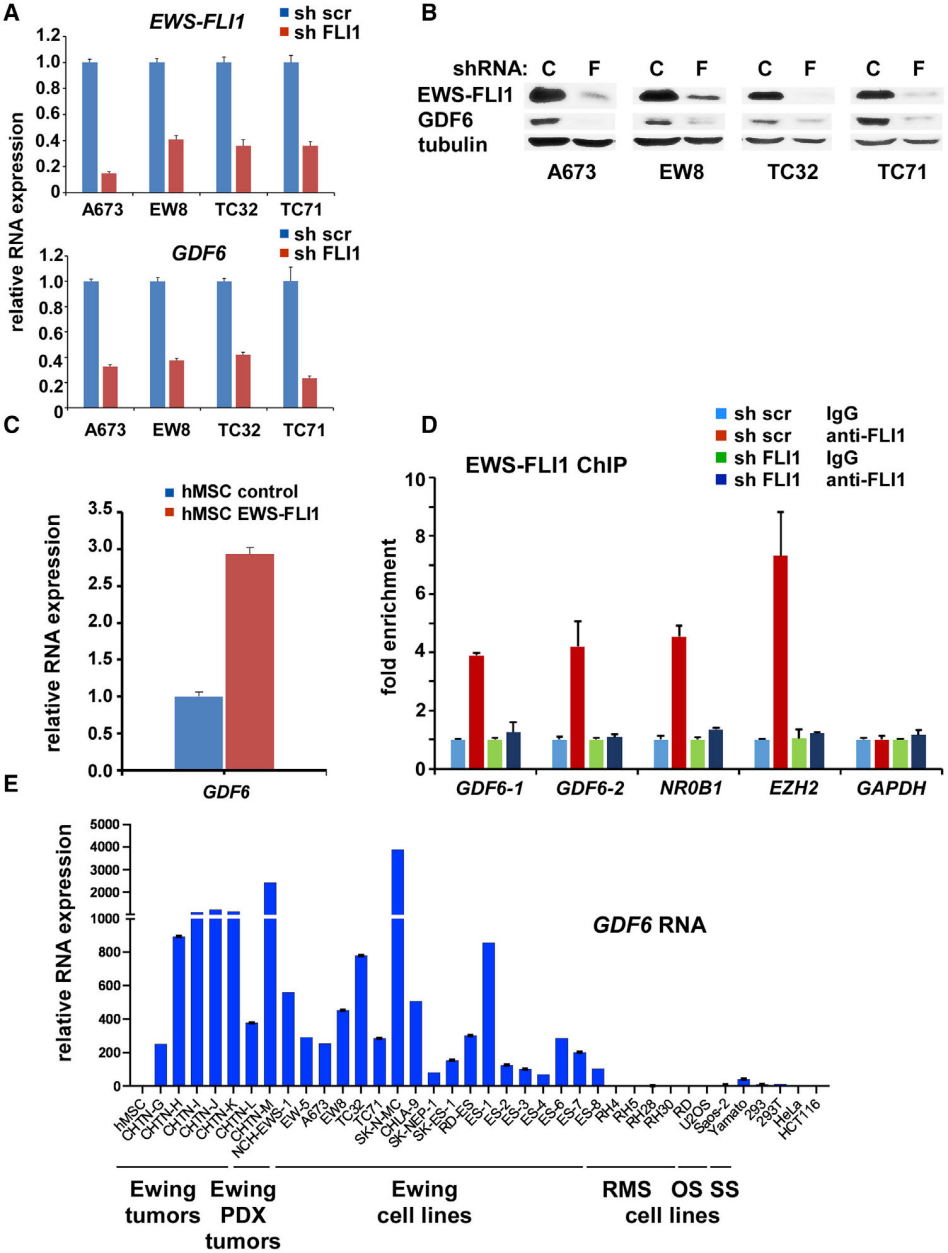
GDF6 Is a Transcriptional Target of EWS-FLI1 and Highly Expressed in Ewing Sarcoma (A) EWS-FLI1 silencing by an shRNA targeting FLI1 C terminus results in reduced *GDF6* RNA expression in Ewing sarcoma (n = 3). (B) EWS-FLI1 silencing results in reduced GDF6 protein expression in Ewing sarcoma. C, control scrambled shRNA; F, FLI1 C-terminal shRNA. (C) EWS-FLI1 induces *GDF6* RNA expression in human mesenchymal stem cells, putative cells of origin of Ewing sarcoma (n = 3). (D) EWS-FLI-1 binds to the *GDF6* gene promoter in Ewing sarcoma. Chromatin immunoprecipitation analysis for EWS-FLI-1 binding to the promoter of *GDF6* and known EWS-FLI1 target genes (*NR0B1* and *EZH2*), as well as control (*GAPDH*), with and without EWS-FLI1 silencing in A673 cells (n = 3). (E) GDF6 is highly expressed in Ewing sarcoma tumors and cell lines. *GDF6* RNA expression was analyzed by quantitative real-time RT-PCR and was normalized to the levels in human mesenchymal stem cells (hMSCs). *GDF6* is highly expressed in Ewing sarcoma tumors, Ewing sarcoma patient-derived xenograft tumors (NCH-EWS-1 and EW-5), and Ewing sarcoma cell lines. (n = 3)

Silencing of EWS-FLI1 by a short hairpin RNA (shRNA) resulted in reduced GDF6 RNA and protein levels in Ewing sarcoma cells (Figures 1A and B). Conversely, lentiviral expression of EWS-FLI1 in human mesenchymal stem cells, putative cells of origin of Ewing sarcoma, increased *GDF6* transcript levels (Figure 1C). By using chromatin immunoprecipitation (ChIP), we detected the binding of EWS-FLI1 to the *GDF6* gene promoter in A673 cells, which was abolished by EWS-FLI1 silencing (Figure 1D). A previous ChIP sequencing study also detected the binding of EWS-FLI1 to the *GDF6* gene in Ewing sarcoma cells (Riggi et al., 2014). These results indicate that *GDF6* is a direct transcriptional activation target of EWS-FLI1. Consistent with *GDF6* being an EWS-FLI1 target, *GDF6* is highly expressed in Ewing sarcoma tumors and cell lines compared with mesenchymal stem cells, rhabdomyosarcoma cells, osteosarcoma cells, synovial sarcoma cells, and epithelial cells, such as 293, 293T, HeLa, and HCT116 (Figure 1E).

### Ewing Sarcoma Depends on the GDF6 Prodomain

**Figure 2.**
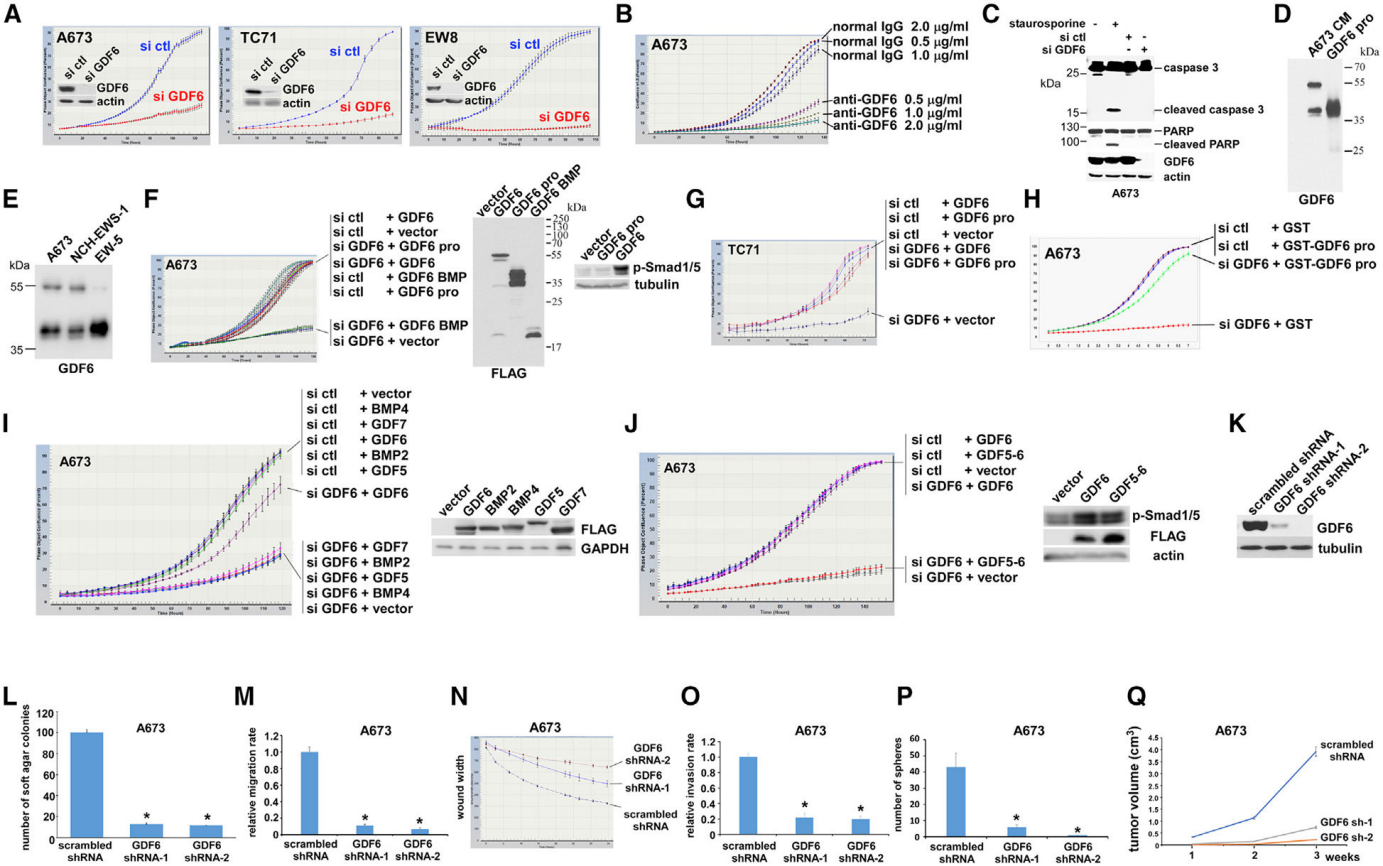
Ewing Sarcoma Depends on the Prodomain of GDF6 (A) GDF6 silencing inhibits Ewing sarcoma proliferation. GDF6 was silenced by siRNA transfection in A673, TC71, and EW8 cells, and cell proliferation was assessed by IncuCyte live-cell imaging system. GDF6 silencing was verified by immunoblotting (inset). (B) Anti-GDF6 prodomain antibody inhibits Ewing sarcoma proliferation. Anti-GDF6 prodomain antibody or control normal IgG was added at the indicated concentration, and cell proliferation was assessed by IncuCyte. (C) GDF6 silencing does not induce apoptosis. GDF6 silencing in A673 cells did not result in the cleavage of caspase-3 and PARP, markers of apoptosis. Staurosporine treatment (1 μM for 4 h) serves as a positive control for apoptosis. (D) A673 cell conditioned medium expresses an approximately 55-kDa precursor and an approximately 40-kDa prodomain of GDF6. The latter comigrates with the GDF6 prodomain secreted from transfected 293T cells. The blot was probed with anti-GDF6 prodomain antibody. (E) Ewing sarcoma patient-derived xenograft tumor cells secrete GDF6. Two patient-derived xenograft tumors (NCH-EWS-1 and EW-5) were dissociated to cells, and the secretion of the GDF6 precursor and the GDF6 prodomain was assessed by anti-GDF6 immunoblotting of the conditioned media. (F) The prodomain, but not the BMP domain, of GDF6 rescues the proliferation inhibition of A673 Ewing sarcoma cells by GDF6 silencing. A673 cells were transfected with GDF6 siRNA or control siRNA and were treated with 293T cell conditioned medium expressing GDF6, GDF6 prodomain, GDF6 BMP domain (N-terminally fused to a signal peptide), or vector. Cell proliferation was assessed by IncuCyte (left). The expression of GDF6, GDF6 prodomain, and GDF6 BMP domain was verified by anti-FLAG immunoblotting (all of these are C-terminally FLAG-tagged; middle). The addition of GDF6, but not GDF6 prodomain conditioned medium, to A673 cells activated BMP signaling assessed by phosphorylated Smad1/5 (right). (G) The GDF6 prodomain rescues the proliferation inhibition of TC71 Ewing sarcoma cells by GDF6 silencing. TC71 cells were transfected with GDF6 siRNA or control siRNA and were treated with 293T cell conditioned medium expressing GDF6, GDF6 prodomain, or vector. Cell proliferation was assessed by IncuCyte. (H) GST-GDF6 prodomain rescues the proliferation inhibition by GDF6 silencing. A673 cells were transfected with GDF6 siRNA or control siRNA and were treated with GST-GDF6 prodomain or GST (500 ng/mL). Cell proliferation was assessed by IncuCyte. (I) Unlike GDF6, GDF5, GDF7, BMP2, and BMP4 are unable to rescue the proliferation inhibition by GDF6 silencing in Ewing sarcoma. A673 cells were transfected with GDF6 siRNA or control siRNA and were treated with 293T cell conditioned medium expressing GDF6, GDF5, GDF7, BMP2, BMP4, or vector. Cell proliferation was assessed by IncuCyte (left). The expression of GDF6, GDF5, GDF7, BMP2, and BMP4 was verified by anti-FLAG immunoblotting (all of these are C-terminally FLAG-tagged; right). (J) Unlike GDF6, the GDF5 prodomain-GDF6 BMP domain fusion protein (GDF5-6) is unable to rescue the proliferation inhibition by GDF6 silencing in Ewing sarcoma. A673 cells were transfected with GDF6 siRNA or control siRNA and were treated with 293T cell conditioned medium expressing GDF5-6, GDF6, or vector. Cell proliferation was assessed by IncuCyte (left). Like GDF6, GDF5-6 was able to activate BMP signaling (right). GDF5-6 and GDF6 are both C-terminally FLAG tagged. (K) shRNA-mediated silencing of GDF6 in A673 cells. A673 cells were infected with lentiviruses expressing shRNAs against GDF6 or control scrambled shRNA. After puromycin selection, GDF6 silencing was verified by immunoblotting. (L) GDF6 silencing impairs anchorage-independent growth of Ewing sarcoma. *p < 0.05 (n = 5). (M) GDF6 silencing reduces the migration rate of Ewing sarcoma assessed by Transwell assays. *p < 0.05 (n = 4). (N) GDF6 silencing reduces the migration rate of Ewing sarcoma assessed by wound closure assays. (O) GDF6 silencing reduces the invasiveness of Ewing sarcoma assessed by Matrigel invasion assays. *p < 0.05 (n = 4). (P) GDF6 silencing inhibits the tumor sphere formation of Ewing sarcoma. *p < 0.05 (n = 5). (Q) GDF6 silencing inhibits the xenograft tumorigenicity of Ewing sarcoma. *p < 0.05 (n = 5).

To clarify the role of GDF6 in Ewing sarcoma, we first tested the effects of manipulation of GDF6 expression. Small interfering RNA (siRNA)-mediated silencing of GDF6 strongly inhibited the proliferation of all 12 Ewing sarcoma cell lines tested (A673, TC71, EW8, ES1, ES2, ES4, SK-N-MC, SK-ES-1, CHLA-258, SK-NEP-1, SK-PNET-Li, and CHLA-352; Figures 2A and S1A). GDF6 siRNA did not affect the proliferation of HeLa and HCT116 cells (Figure S1B). Ewing sarcoma growth was also inhibited by anti-GDF6 antibody in a dose-dependent manner (Figure 2B). Although GDF6 downregulation strongly inhibits Ewing sarcoma growth, this was not accompanied by the induction of apoptosis (Figure 2C). The BMP family proteins are synthesized as a precursor molecule with the prodomain at the N terminus and the BMP domain at the C terminus; proteolytic cleavage at the RXXR cleavage site separates the two domains in Golgi (Aono et al., 1995; Cui et al., 1998). Using anti-GDF6 prodomain immunoblotting, we detected an approximately 55-kDa precursor and an approximately 40-kDa prodomain in the A673 cell conditioned medium (Figure 2D, lane 1). The latter co-migrated with the GDF6 prodomain expressed in 293T cells (Figure 2D, lane 2). The secretion of the GDF6 precursor and the GDF6 prodomain was also detected in dissociated cells from Ewing sarcoma patient-derived xenograft tumors (Figure 2E).

The growth inhibition by GDF6 silencing was completely rescued by the addition of conditioned medium from GDF6-transfected 293T cells (Figures 2F and 2G), indicating that Ewing sarcoma depends on extracellular GDF6. Surprisingly, the GDF6 prodomain conditioned medium, but not the GDF6 BMP domain conditioned medium, was also able to rescue the growth inhibition by GDF6 silencing (Figures 2F and 2G), suggesting that Ewing sarcoma depends on the GDF6 prodomain. The GDF6 prodomain did not stimulate BMP signaling in Ewing sarcoma, whereas full-length GDF6 did (Figure 2F, right; BMP signaling assessed by the levels of phosphorylated Smad1/5). Furthermore, purified recombinant GST-GDF6 prodomain produced in bacteria rescued the growth inhibition by GDF6 silencing (Figure 2H). The BMP domain of GDF6 displays high homology (56%–82%) with GDF5, GDF7, BMP2, and BMP4, whereas the GDF6 prodomain is more divergent (Williams et al., 2008). We found that unlike GDF6, GDF5, GDF7, BMP2, and BMP4 are unable to rescue the growth inhibition by GDF6 silencing (Figure 2I). Additionally, we assessed the activity of a fusion protein with the GDF5 prodomain fused to the GDF6 BMP domain (GDF5-6). Although GDF5-6 and GDF6 were both able to simulate BMP signaling (Figure 2J, right), unlike GDF6, GDF5-6 was unable to rescue the growth inhibition by GDF6 silencing (Figure 2J, left), further supporting the critical role for the GDF6 prodomain in Ewing sarcoma.

The dependence of Ewing sarcoma on the prodomain, but not the BMP domain, of GDF6 prompted us to further test the role of BMP signaling in Ewing sarcoma. We employed a secreted BMP inhibitor, noggin, which binds and inactivates BMPs (Krause et al., 2011). Treatment of Ewing sarcoma cells with noggin eliminated phosphorylated Smad1/5 (Figure S2A). This indicates that noggin efficiently blocked BMP signaling in Ewing sarcoma cells. However, noggin treatment had no effect on the proliferation of Ewing sarcoma cells (Figure S2B), indicating that Ewing sarcoma does not depend on BMP signaling.

We then used lentivirus-mediated shRNA expression to stably silence GDF6 (Figure 2K). shRNA-mediated silencing of GDF6 severely impaired anchorage-independent growth (Figure 2L), migration (Transwell assays in Figure 2M and wound closure assays in Figure 2N), invasion (Figure 2O), sphere formation (Figure 2P), and xenograft tumorigenicity (Figure 2Q) of Ewing sarcoma cells. These results indicate that Ewing sarcoma depends on GDF6, an EWS-FLI1 target, and that the GDF6 prodomain mediates the critical growth signaling in Ewing sarcoma.

### CD99 Serves as the Receptor for the GDF6 Prodomain

CD99 is a transmembrane protein highly expressed in Ewing sarcoma that has been widely used as a diagnostic marker to distinguish Ewing sarcoma and other small round blue cell tumors (Ambros et al., 1991; Fellinger et al., 1991; Kovar et al., 1990). In addition, although the molecular mechanism is not well understood, there is evidence that CD99 contributes to Ewing sarcoma growth: CD99 knockdown in Ewing sarcoma cells inhibited anchorage-dependent and anchorage-independent growth, and reduced tumorigenicity (Rocchi et al., 2010). Because the phenotype of CD99-silenced Ewing sarcoma cells resembles that of GDF6-silenced Ewing sarcoma cells, we tested the possibility that CD99 is the GDF6 prodomain receptor.

**Figure 3.**
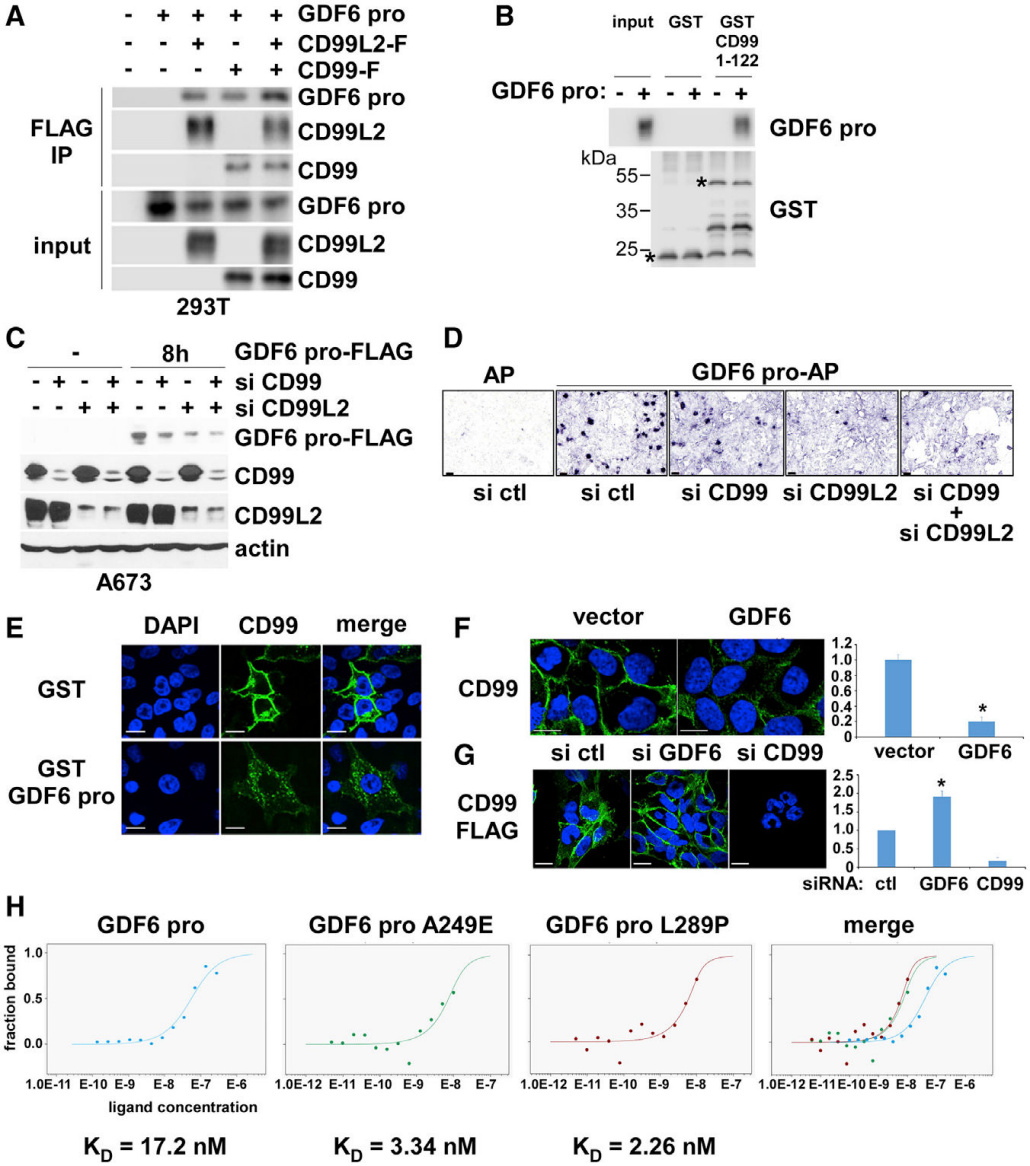
CD99 Serves as the GDF6 Prodomain Receptor in Ewing Sarcoma (A) GDF6 prodomain co-immunoprecipitates with CD99 and CD99L2, a paralog of CD99, upon co-transfection in 293T cells. C-terminally FLAG-tagged CD99 and/or CD99L2 was co-transfected with GDF6 prodomain in 293T cells. The interaction between GDF6 prodomain and CD99/CD99L2 was assessed by anti-FLAG immunoprecipitation followed by immunoblotting for the indicated protein. (B) GDF6 prodomain interacts with GST-CD99(1–122) *in vitro*. GDF6 prodomain expressed in 293T cell conditioned medium was incubated with GST-CD99 extracellular domain (1–122) or GST, and the protein interaction was assessed by GST pull-down followed by immunoblotting. Asterisks denote GST-CD99(1–122) and GST. (C) GDF6 prodomain binds to Ewing sarcoma cells through CD99/CD99L2. C-terminally FLAG-tagged GDF6 prodomain secreted from transfected 293T cells was incubated for 8 h with A673 cells that were transfected with CD99 siRNA, CD99L2 siRNA, and control siRNA as indicated (“–” denotes controls without incubation). Cells were washed, and the whole-cell lysate was prepared to assess the binding of GDF6 prodomain-FLAG to the cells. GDF6 prodomain-FLAG bound to control siRNA-transfected cells, and the binding was reduced by CD99 and CD99L2 silencing. The silencing of CD99 and CD99L2 was verified by immunoblotting. (D) CD99/CD99L2 mediate the binding of GDF6 prodomain to Ewing sarcoma cells. GDF6 prodomain fused to alkaline phosphatase (AP), or AP alone was produced from transfected 293T cells and was incubated with A673 cells that were transfected with CD99 siRNA and/or CD99L2 siRNA or control siRNA as indicated. The binding of GDF6 prodomain-AP or AP to cells was visualized by AP reaction. GDF6 prodomain-AP bound to control siRNA-expressing A673 cells, and the binding was blocked by CD99 siRNA and CD99L2 siRNA. Scale bars: 20 μm. (E) GDF6 prodomain induces the internalization of CD99 expressed in HeLa cells. CD99 expressed in HeLa cells was located in the plasma membrane. GST-GDF6 prodomain caused the internalization of CD99. Scale bars: 20 μm. (F) GDF6 induces the internalization of endogenous CD99 in A673 cells. A673 cells were treated with 293T cell conditioned medium expressing vector or GDF6. The latter induced the internalization of endogenous CD99 detected by anti-CD99 immunofluorescence (left). Scale bars: 20 μm. The quantification of surface CD99 expression (right, *p < 0.05; n = 5). (G) GDF6 silencing results in accumulation of CD99 on A673 cell surface. A673 stably expressing C-terminally FLAG-tagged CD99 was transfected with control siRNA or GDF6 siRNA, and the location of CD99-FLAG was assessed by anti-FLAG immunostaining (left). Scale bars: 20 μm. GDF6 silencing caused the accumulation of CD99-FLAG on cell surface. CD99 siRNA abolished the immunostaining of CD99-FLAG. The quantification of surface CD99-FLAG expression (right, *p < 0.05; n = 5). (H) Microscale thermophoresis analysis of GDF6 prodomain-CD99 extracellular domain interaction. The interaction between C-terminally Fc-tagged CD99 extracellular domain (residues 23–122) and GDF6 prodomain (wild-type, A249E, or L289P) was analyzed by microscale thermophoresis. GDF6 prodomain mutants displayed lower K_D_ values than wild-type GDF6 prodomain.

The GDF6 prodomain expressed in 293T cells co-immunoprecipitated with CD99, as well as CD99L2, a paralog of CD99 (Figure 3A). The interaction between GDF6 prodomain and CD99 was also verified by the pull-down of GDF6 prodomain conditioned medium with the GST fusion of CD99 extracellular domain (Figure 3B).

We then used ligand-binding assays to test the role of CD99 and CD99L2 as the GDF6 prodomain receptor in Ewing sarcoma. C-terminally FLAG-tagged GDF6 prodomain (GDF6 pro-FLAG) was produced from transfected 293T cells and incubated with A673 cells that were transfected with CD99 siRNA and/or CD99L2 siRNA or control siRNA. After washing the cells to remove unbound GDF6 pro-FLAG, a whole cell lysate was prepared and analyzed by immunoblotting to assess the binding of GDF6 pro-FLAG to A673 cells. As shown in Figure 3C, GDF6 pro-FLAG bound to control siRNA-transfected A673 cells, and this binding was significantly reduced by silencing of CD99 and/or CD99L2, indicating that the GDF6 prodomain binds CD99/CD99L2 in Ewing sarcoma. The specificity of the GDF6 prodomain-CD99/CD99L2 interaction was further confirmed by ligand-binding assays using alkaline phosphatase-fused GDF6 prodomain (GDF6 pro-AP) (Figure 3D). GDF6 pro-AP bound efficiently to A673 cells transfected with control siRNA, but the binding was diminished in A673 cells transfected with CD99 siRNA and/or CD99L2 siRNA (Figure 3D).

CD99 expressed by transfection in HeLa cells was located in the cell membrane, and recombinant GST-GDF6 prodomain induced the internalization of CD99 (Figure 3E). Similarly, treatment of A673 cells with GDF6 conditioned medium caused the internalization of endogenous CD99 (Figure 3F). Conversely, silencing of GDF6 resulted in increased surface expression of CD99 in A673 cells (Figure 3G). These results suggest that the binding of GDF6 to CD99 results in the internalization of CD99.

We employed microscale thermophoresis to further assess the interaction between GDF6 prodomain and CD99, and found the KD of GDF6 prodomain-CD99 extracellular domain interaction to be 17.2 nM (Figure 3H, left).

### GDF6-CD99 Signaling Downregulates Src through CSK

**Figure 4.**
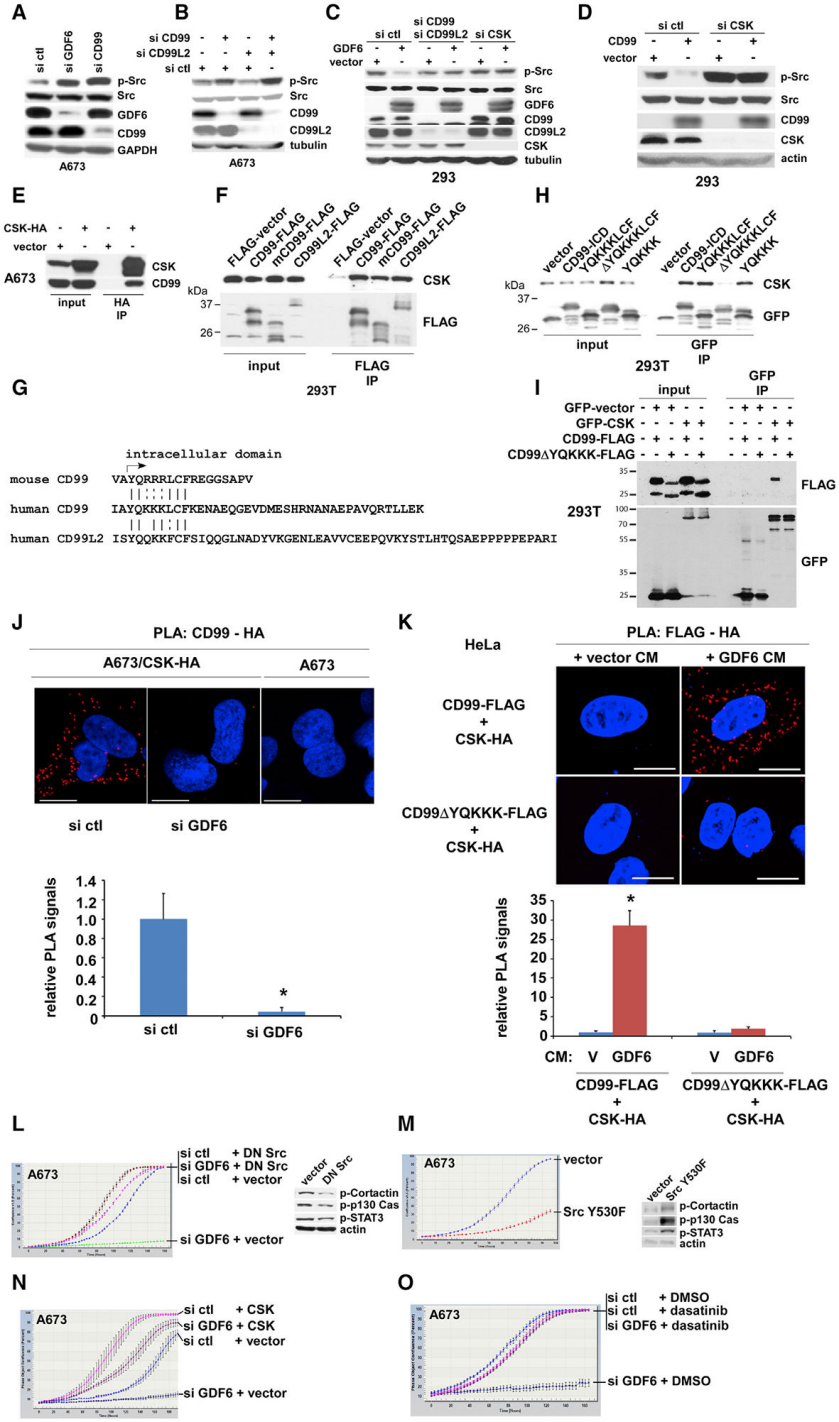
GDF6 Prodomain-CD99 Signaling Inhibits Src through Recruitment of CSK to the YQKKK Motif in the Intracellular Domain of CD99 (A) GDF6 silencing or CD99 silencing activates Src in A673 cells. Activation of Src was assessed by phosphorylation of tyrosine 419. (B) Silencing of CD99 and CD99L2 activates Src in A673 cells. (C) Inhibition of Src by GDF6 requires CD99/CD99L2 and CSK. Transfection of 293 cells with GDF6 inhibited Src (lanes 1 and 2), which was abolished by CD99 and CD99L2 silencing (lanes 3 and 4) or by CSK silencing (lanes 5 and 6). (D) Inhibition of Src by CD99 requires CSK. Transfection of 293 cells with CD99 inhibited Src (lanes 1 and 2), which was abolished by CSK silencing (lanes 3 and 4). (E) CSK co-immunoprecipitates with CD99. C-terminally HA-tagged CSK was stably expressed in A673 cells and was immunoprecipitated with anti-HA antibody. The co-precipitation of endogenous CD99 was assessed by immunoblotting. (F) Human CD99, mouse CD99, and human CD99L2 co-immunoprecipitate with CSK. C-terminally FLAG-tagged human CD99, mouse CD99, and human CD99L2 were transfected in 293T cells, and the interaction with endogenous CSK was assessed by anti-FLAG immunoprecipitation followed by anti-CSK immunoblotting. Immunoprecipitation of each FLAG-tagged protein was verified by anti-FLAG immunoblotting. (G) Conserved YQKKKLCF-like motifs in the juxtamembrane regions of human CD99, mouse CD99, and human CD99L2. (H) The YQKKK motif interacts with CSK. Human CD99 intracellular domain (148–185), human CD99 intracellular domain lacking the YQKKKLCF sequence, YQKKKLCF, or YQKKK (all N-terminally fused to GFP) was transfected in 293T cells, and the interaction with endogenous CSK was assessed by anti-GFP immunoprecipitation followed by anti-CSK immunoblotting. Immunoprecipitation of each GFP-fusion protein was verified by anti-GFP immunoblotting (bottom). (I) The deletion of the YQKKK motif abrogates CD99-CSK interaction. C-terminally FLAG-tagged CD99 or CD99 lacking the YQKKK motif was co-transfected with GFP-tagged CSK or empty GFP vector in 293T cells. The CD99-CSK interaction was assessed by anti-GFP immunoprecipitation followed by anti-FLAG immunoblotting. (J) Endogenous GDF6 stimulates CD99-CSK interaction in Ewing sarcoma. The proximity ligation assays (PLAs) were used to assess the recruitment of CSK to CD99 in A673 cells stably expressing C-terminally HA-tagged CSK. The robust PLA signals were detected between CD99 and CSK-HA, which were abolished by GDF6 silencing. Scale bars: 20 μm. The quantification of PLA signals is shown at the bottom. *p < 0.05 (n = 5). (K) GDF6 stimulates the CSK recruitment to the YQKKK motif in CD99. HeLa cells, which do not endogenously express GDF6, were transfected with CD99-FLAG and CSK-HA and then treated with 293T cell conditioned medium expressing vector or GDF6, and the interaction between CD99-FLAG and CSK-HA was assessed by the PLA (top). GDF6 conditioned medium induced the recruitment of CSK-HA to CD99 (top). The same assay was performed using CD99 lacking the YQKKK motif, which showed that GDF6 conditioned medium was unable to induce the recruitment of CSK-HA to CD99 lacking the YQKKK motif (middle). Scale bars: 20 μm. The quantification of PLA signals is shown at the bottom. *p < 0.05 (n = 5). (L) Dominant-negative Src abrogates the proliferation inhibition by GDF6 silencing in A673 cells. A673 cells stably expressing dominant-negative Src or empty vector were transfected with GDF6 siRNA or control siRNA, and cell proliferation was assessed by IncuCyte (left). Dominant-negative Src reduced the phosphorylation of Src substrates, cortactin, p130 Cas, and STAT3 (right). (M) Src mutant lacking the CSK phosphorylation site (Y530F) inhibits A673 cell proliferation. Proliferation of A673 cells stably expressing Src Y530F or empty vector was assessed by IncuCyte (left). Src Y530F increased the phosphorylation of Src substrates, cortactin, p130 Cas, and STAT3 (right). (N) CSK abrogates the proliferation inhibition by GDF6 silencing in A673 cells. A673 cells stably expressing CSK or empty vector were transfected with GDF6 siRNA or control siRNA, and cell proliferation was assessed by IncuCyte. (O) A Src inhibitor, dasatinib, abrogates the proliferation inhibition by GDF6 silencing in A673 cells. Dasatinib was used at 50 nM.

CD99 has been shown to inhibit Src by an unknown mechanism in osteosarcoma (Scotlandi et al., 2007) and acute myeloid leukemia (Chung et al., 2017). We, therefore, investigated the effect of manipulation of the levels of GDF6 and CD99/CD99L2 on Src activity. Silencing of GDF6 or CD99 in Ewing sarcoma cells resulted in increased levels of active Src (Figure 4A, active Src assessed by phosphorylation of tyrosine 419 in human Src). Simultaneous silencing of CD99 and CD99L2 resulted in further increase in active Src levels (Figure 4B, lanes 2 and 4). Conversely, transfection of GDF6 in 293 cells reduced active Src levels (Figure 4C, lanes 1 and 2). Importantly, GDF6 was unable to inhibit Src when endogenous CD99 and CD99L2 were silenced by siRNA transfection (Figure 4C, lanes 3 and 4), suggesting that GDF6 inhibits Src through CD99/CD99L2.

The activity of Src is restricted by CSK, which phosphorylates the C-terminal regulatory tyrosine in Src (tyrosine 530 in human Src) and inactivates its activity (Okada, 2012). The inhibition of Src by CSK is thought to be regulated by the recruitment of CSK to the inner face of plasma membrane, where Src is located (Okada, 2012). Several proteins have been identified to serve as the plasma membrane anchor for CSK, including caveolin-1 (Cao et al., 2002), paxillin (Sabe et al., 1994), VE-cadherin (Baumeister et al., 2005), IGF-1R (Arbet-Engels et al., 1999), insulin receptor (Arbet-Engels et al., 1999), LIME (Brdicková et al., 2003), SIT (Pfrepper et al., 2001), and Cbp/PAG (Brdicka et al., 2000; Kawabuchi et al., 2000). We found that GDF6 is not able to inhibit Src when CSK is silenced (Figure 4C, lanes 5 and 6). Furthermore, silencing of CSK also abolished CD99-mediated inhibition of Src in 293 cells (Figure 4D). These results suggest that GDF6 and CD99 inhibit Src through CSK.

Because of the lack of an appropriate anti-CSK antibody for immunoprecipitation, we stably expressed C-terminally hemag-glutinin (HA)-tagged CSK in A673 and assessed the interaction between CSK and CD99 by anti-HA immunoprecipitation, which detected the co-immunoprecipitation of endogenous CD99 with CSK-HA (Figure 4E). Conversely, C-terminally FLAG-tagged human CD99, mouse CD99, and human CD99L2 co-immuno-precipitated with endogenous CSK in 293T cells (Figure 4F). Comparison of the intracellular domains of human CD99, mouse CD99, and human CD99L2 revealed conserved YQKKKLCF-like motifs in the juxtamembrane regions (Figure 4G). When fused to GFP, human CD99 intracellular domain, as well as the YQKKKLCF sequence, was able to co-immunoprecipitate with endogenous CSK in 293T cells, whereas human CD99 intracellular domain lacking the YQKKKLCF sequence was unable to interact with CSK (Figure 4H). Furthermore, the YQKKK sequence fused to GFP was sufficient for interaction with CSK (Figure 4H). C-terminally FLAG-tagged CD99 co-immunoprecipitated with GFP-tagged CSK in 293T cells; the deletion of the YQKKK motif in CD99 abolished CSK interaction (Figure 4I). These results suggest that the YQKKK motif mediates the interaction with CSK.

We employed proximity ligation assays (PLAs) to further assess the interaction between CD99 and CSK in cells. Using A673 cells stably expressing CSK-HA, robust PLA signals (red dots) between CD99 and CSK-HA were observed, and importantly, GDF6 silencing abolished the CD99-CSK-HA PLA signals (Figure 4J). This suggests that endogenous GDF6 stimulates the interaction between CD99 and CSK in Ewing sarcoma. Conversely, GDF6 conditioned medium dramatically increased the PLA signals between CD99 and CSK-HA in CD99- and CSK-HA-co-transfected HeLa cells (Figure 4K, top), which do not endogenously express GDF6. This further supports the role of GDF6 in stimulating CD99-CSK interaction. GDF6 conditioned medium was not able to increase the PLA signals between YQKKK-deleted CD99 and CSK-HA (Figure 4K, bottom), which is consistent with the requirement of the YQKKK motif for CSK recruitment to CD99.

We then evaluated the role of Src in the regulation of Ewing sarcoma growth by GDF6-CD99 signaling. Lentiviral expression of dominant-negative Src reduced the tyrosine phosphorylation of Src substrates, cortactin, p130 Cas, and STAT3 (Figure 4L, right) and abrogated growth inhibition by the silencing of GDF6 (Figure 4L, left). Conversely, the expression of Src with a mutation in the CSK phosphorylation site (Y530F), which evades inactivation by CSK, inhibited Ewing sarcoma growth (Figure 4M). Growth inhibition by GDF6 silencing was also abolished by exogenous expression of CSK (Figure 4N) and by a Src inhibitor, dasatinib (Figure 4O). These results suggest that GDF6 silencing inhibits Ewing sarcoma growth by inducing hyperactivation of Src.

We also assessed the role of GDF6-CD99-Src signaling in migration of Ewing sarcoma cells. Inhibition of cell migration by GDF6 silencing was abolished by dominant-negative Src and by CSK (Figure S3), suggesting that GDF6 silencing inhibits Ewing sarcoma migration through hyperactivation of Src. A study in osteosarcoma demonstrated that CD99 exogenous expression inhibits cell migration through inhibition of Src and Rock2 (Zucchini et al., 2014). The opposing activities of Src detected in the osteosarcoma study and the present study may reflect the different sarcoma types or different experimental conditions employed (CD99 exogenous expression in osteosarcoma versus endogenous GDF6 silencing in Ewing sarcoma).

GDF6 silencing in Ewing sarcoma resulted in induction of a CDK inhibitor, p21 (Figure S4A). Growth arrest induced by GDF6 silencing in Ewing sarcoma was abolished by simultaneous silencing of p21 (Figure S4B), indicating that GDF6 silencing results in p21-dependent growth arrest. Although mitogen-activated protein kinase (MAPK) was shown to be activated by CD99 silencing in Ewing sarcoma (Rocchi et al., 2010), GDF6 silencing still induced p21 when the MAPK pathway was suppressed by a MEK inhibitor, PD98059 (Figure S4C). Dominant-negative Src, which abolished growth arrest by GDF6 silencing (Figure 4L), also abolished p21 induction by GDF6 silencing (Figure S4D). Similarly, exogenous expression of CSK, which abolished growth arrest by GDF6 silencing (Figure 4N), also abolished p21 induction by GDF6 silencing (Figure S4E). Furthermore, dasatinib, which abolished growth arrest by GDF6 silencing (Figure 4O), also abolished p21 induction by GDF6 silencing (Figure S4F). Conversely, Src Y530F mutant, which inhibited Ewing sarcoma growth (Figure 4M), induced p21 (Figure S4G). Furthermore, CSK silencing in Ewing sarcoma resulted in activation of Src (Figure S4H), induction of p21 (Figure S4H), and growth arrest (Figure S4I). Collectively, these results are consistent with the notion that GDF6 silencing causes p21-dependent growth arrest of Ewing sarcoma through Src hyperactivation.

### GDF6 Prodomain Mutants Associated with Klippel-Feil Syndrome Are Hyperactive in GDF6-CD99-Src Signaling

Mutations in the prodomain of GDF6 are associated with Klippel-Feil syndrome, a congenital malformation syndrome characterized by the fusion of cervical vertebrae and variable abnormalities in multiple organs (Tassabehji et al., 2008). Two recurrent GDF6 prodomain mutations in Klippel-Feil syndrome patients are A249E and L289P (Tassabehji et al., 2008). GDF6 A249E mutation is also linked to microphthalmia/anophthalmia with and without skeletal abnormalities (Asai-Coakwell et al., 2009; den Hollander et al., 2010; Gonzalez-Rodriguez et al., 2010; Slavotinek et al., 2015), hereditary retinal dystrophy (Asai-Coakwell et al., 2013), and Chiari type I malformation (displacement of the cerebellar tonsils below the base of the skull) (Markunas et al., 2013). In addition, several other GDF6 prodomain mutations were associated with congenital eye and skeletal abnormalities (Asai-Coakwell et al., 2009; Asai-Coakwell et al., 2013; Chassaing et al., 2014; den Hollander et al., 2010; Gonzalez-Rodriguez et al., 2010).

The pathogenic role of GDF6 prodomain mutations is not well understood. GDF6 prodomain mutants were reported to display diminished BMP signaling activity (Asai-Coakwell et al., 2013), which is not consistent with the autosomal dominant inheritance of abnormalities associated with GDF6 prodomain mutations. We thus evaluated the effects of GDF6 A249E and L289P mutations on GDF6-CD99-Src signaling.

**Figure 5.**
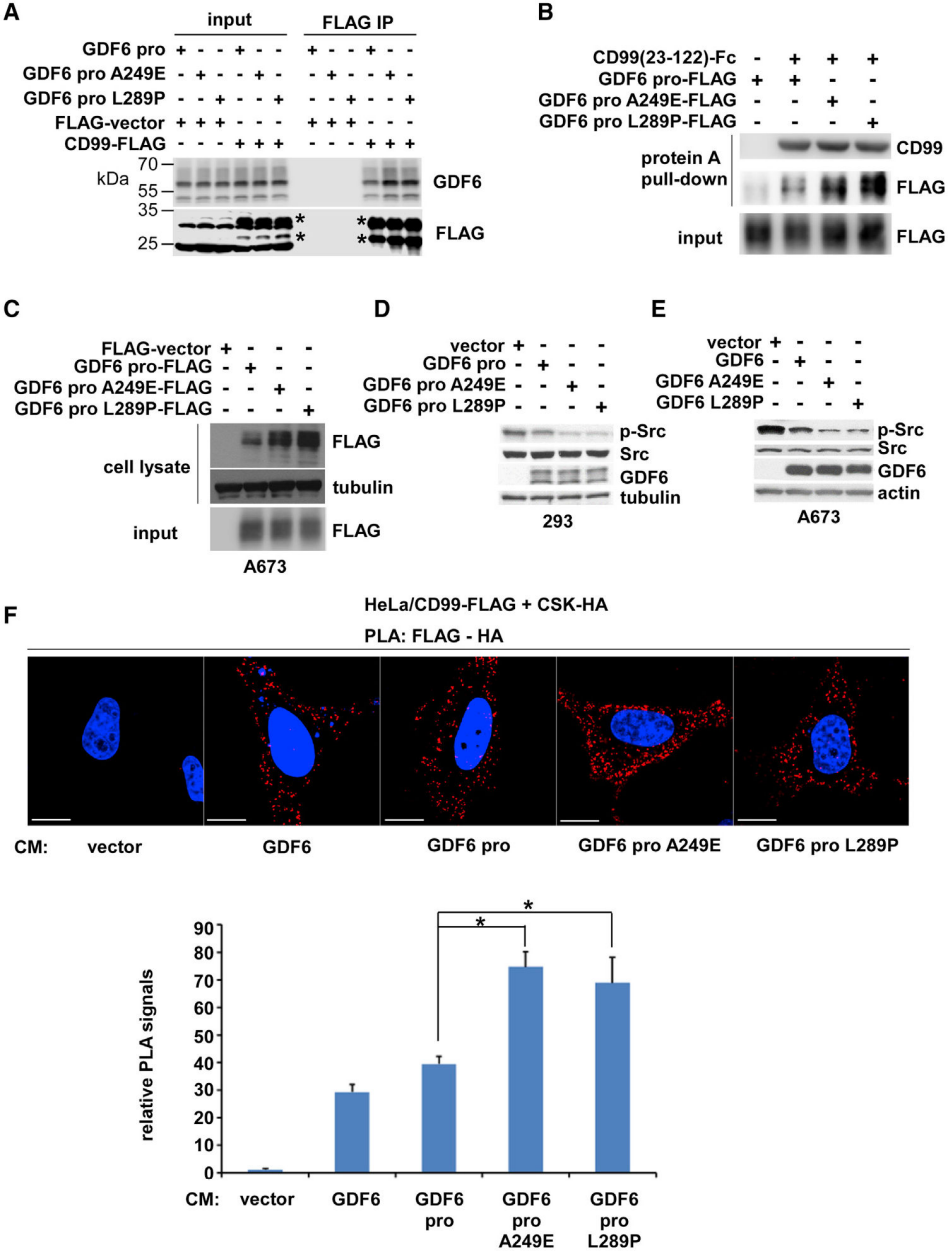
GDF6 Prodomain Mutants Associated with Klippel-Feil Syndrome Are Hyperactive in GDF6-CD99-Src Signaling (A) GDF6 A249E or L289P prodomain mutant displays enhanced co-immunoprecipitation with CD99 compared with wild-type GDF6 prodomain. GDF6 prodomain, GDF6 A249E prodomain, or GDF6 L289P prodomain was co-transfected with C-terminally FLAG-tagged CD99 or FLAG vector in 293T cells as indicated, and the interaction between each GDF6 prodomain and CD99-FLAG was assessed by anti-FLAG immunoprecipitation followed by immunoblotting. (B) GDF6 A249E or L289P prodomain mutant displays enhanced interaction with CD99 *in vitro*. Wild-type, A249E, or L289P GDF6 prodomain expressed in 293T cell conditioned medium was incubated with C-terminally Fc-tagged CD99 extracellular domain (residues 23–122), and protein interaction was assessed by protein A Sepharose pull-down followed by immunoblotting. (C) GDF6 A249E or L289P prodomain mutant displays enhanced binding to A673 cells. Ligand-binding assays were performed as in Figure 3C. (D) GDF6 A249E or L289P prodomain mutant more strongly inhibits Src than wild-type prodomain in 293 cells. Wild-type, A249E, or L289P GDF6 prodomain was stably expressed in 293 cells, and the levels of active Src (phosphorylated on Y419) were assessed by immunoblotting. (E) GDF6 A249E or L289P mutant more strongly inhibits Src than wild-type GDF6 in A673 cells. Wild-type, A249E, or L289P GDF6 was stably expressed in A673 cells, and the levels of active phosphorylated Src were assessed by immunoblotting. (F) GDF6 A249E or L289P prodomain mutant more strongly induces CSK recruitment to CD99. The PLA was performed in HeLa cells as in Figure 4K, using 293T cell conditioned medium expressing vector, GDF6, GDF6 prodomain, GDF6 A249E prodomain, or GDF6 L289P prodomain. Scale bars: 20 μm. The quantification of PLA signals is shown at the bottom. *p < 0.05 (n = 5).

As demonstrated in Figure 3A, GDF6 prodomain co-immunoprecipitates with CD99 upon co-transfection in 293T cells; GDF6 A249E and L289P prodomains displayed enhanced co-immunoprecipitation with CD99 compared with wild-type prodomain (Figure 5A). GDF6 A249E and L289P prodomains also displayed enhanced interaction with CD99 by *in vitro* binding assays: after incubation *in vitro*, Fc-tagged CD99 extracellular domain (23–122) pulled down more GDF6 A249E and L289P prodomains than wild-type GDF6 prodomain (Figure 5B). Ligand-binding assays revealed that GDF6 A249E and L289P prodomains display increased binding to A673 cells compared with wild-type GDF6 prodomain (Figure 5C). Microscale thermophoresis analysis determined the KD of GDF6 A249E and L289P prodomain interaction with CD99 to be 3.34 and 2.26 nM, respectively, which are much lower than the K_D_ for wild-type GDF6 prodomain (17.2 nM) (Figure 3H). Lentiviral expression of GDF6 A249E and L289P prodomains more efficiently inhibited Src than did wild-type GDF6 prodomain in 293 cells (Figure 5D). Similarly, lentiviral expression of GDF6 A249E and L289P mutants more efficiently inhibited Src than wild-type GDF6 in A673 cells (Figure 5E). GDF6 and GDF6 prodomain conditioned media were able to increase the PLA signals between CD99 and CSK-HA in co-transfected HeLa cells (Figure 5F); GDF6 A249E and L289P prodomain conditioned media more strongly enhanced the PLA signals between CD99 and CSK-HA (Figure 5F). These results suggest that GDF6 prodomain mutants linked to Klippel-Feil syndrome are hyperactive in GDF6-CD99-Src signaling.

## DISCUSSION

In this study, we uncovered a cytokine signaling pathway that controls the growth of Ewing sarcoma. We identified *GDF6* as a transcriptional target of EWS-FLI1 (Figure 1) and determined that Ewing sarcoma depends on the autocrine signaling mediated by GDF6 (Figure 2). Surprisingly, we found that Ewing sarcoma depends on the prodomain, not the BMP domain, of GDF6 (Figure 2), and demonstrated that the GDF6 prodomain uses CD99 as a cell surface receptor to inhibit Src (Figures 3 and 4). Mechanistically, we determined that the binding of GDF6 prodomain to CD99 results in recruitment of CSK to the YQKKK motif in the juxtamembrane region of CD99, leading to Src inactivation (Figure 4).

CD99 is highly expressed in Ewing sarcoma and has been used as a marker for this cancer (Ambros et al., 1991; Fellinger et al., 1991; Kovar et al., 1990). CD99 silencing inhibits *in vitro* and *in vivo* growth of Ewing sarcoma (Kreppel et al., 2006; Rocchi et al., 2010). Additionally, CD99 was shown to inhibit Src by an unknown mechanism in osteosarcoma (Scotlandi et al., 2007) and acute myeloid leukemia (Chung et al., 2017). This study revealed that CD99 functions as a signaling receptor, recruiting CSK and inactivating Src in response to GDF6 prodomain (Figures 3 and 4). It will be interesting to determine whether a similar signaling mechanism plays a role in other reported functions for CD99, such as trans-endothelial migration of leukocytes (Schenkel et al., 2002).

CD99 plays a dual role in cancer (Manara et al., 2018). Whereas the silencing of endogenous CD99 revealed a tumor-promoting role in Ewing sarcoma (Kreppel et al., 2006; Rocchi et al., 2010), exogenous expression of CD99 uncovered a tumor-suppressing role in osteosarcoma (Manara et al., 2006; Sciandra et al., 2014; Scotlandi et al., 2007; Zucchini et al., 2014). In osteosarcoma, exogenous CD99 was shown to inhibit osteosarcoma cell migration through inhibition of Src and Rock2 (Zucchini et al., 2014). In Ewing sarcoma, by contrast, endogenous GDF6-CD99 signaling stimulates growth and migration by preventing Src hyperactivation (Figures 4 and S3). It is plausible that the CD99-Src axis plays a dual role depending on cellular context. Understanding how the CD99-Src axis functions in different cell types will likely provide an important insight into the “oncojanus” role for CD99 (Manara et al., 2018).

A Src inhibitor, dasatinib, was previously shown to inhibit Ewing sarcoma growth (Berning et al., 2018; Timeus et al., 2008), although these studies used higher doses of dasatinib than the dose used to detect the rescue of growth arrest by GDF6 silencing (Figure 4O, dasatinib used at 50 nM). Dasatinib was also shown to suppress microenvironmental stress-induced migration and tenascin expression in Ewing sarcoma (Bailey et al., 2016; Hawkins et al., 2019). A phase II clinical trial of dasatinib against a variety of sarcomas, including Ewing sarcoma, concluded that dasatinib is inactive as a single agent (Schuetze et al., 2016). The lack of clinical efficacy of dasatinib in Ewing sarcoma patients is in line with the tumor-promoting role for the prevention of Src hyperactivation by GDF6-CD99 signaling in Ewing sarcoma.

This study also uncovered that the GDF6 prodomain functions as a secreted signaling molecule: a ligand for CD99 that downregulates Src by inducing CSK recruitment to CD99 (Figures 3 and 4). Although the prodomain of BMP family proteins has been shown to play a role in the proper folding and activity of the BMP domain (Neugebauer et al., 2015), the GDF6 prodomain without the BMP domain was sufficient to rescue growth arrest by GDF6 silencing (Figures 2F-2H), indicating the critical role of the BMP-independent prodomain function in Ewing sarcoma. This was further supported by that abrogation of BMP signaling by Noggin had no effect on Ewing sarcoma growth (Figure S2).

Therefore, the *GDF6* gene encodes two bioactive polypeptides that regulate two distinct signaling pathways: the CD99-CSK-Src pathway by the GDF6 prodomain and the BMP-Smad pathway by the GDF6 BMP domain. Although the data presented in this study suggest that GDF6 prodomain signaling, not GDF6 BMP signaling, plays a critical role in established Ewing sarcoma, it is possible that *GDF6* gene expression results in coordinated regulation and crosstalk of GDF6 prodomain signaling and GDF6 BMP signaling in other biological context(s), which may include the initiation phase of Ewing sarcoma.

Our data also suggest that the GDF6 prodomain mutants associated with Klippel-Feil syndrome, A249E and L289P, are hyperactive in GDF6 prodomain signaling (Figure 5). Compared with wild-type GDF6, these GDF6 prodomain mutants bind more tightly to CD99, recruit CSK to CD99 more efficiently, and inhibit Src more potently. These results suggest that the Klippel-Feil syndrome-linked GDF6 mutants display a gain-of-function activity, which is consistent with the autosomal dominant inheritance of GDF6-linked Klippel-Feil syndrome. Interestingly, the GDF6 prodomain mutants associated with Klippel-Feil syndrome have not been reported in Ewing sarcoma, which may suggest that although the presence of GDF6 prodomain signaling is critical in Ewing sarcoma (Figure 2), the intensity of GDF6 prodomain signaling is not critical. This hypothesis is consistent with the lack of correlation between GDF6 expression levels in Ewing sarcoma tumors and patient survival (Figure S5). In contrast, the intensity of GDF6 prodomain signaling may play a critical role in the pathogenesis of Klippel-Feil syndrome. It will be important in the future to determine how hyperactive GDF6 prodomain signaling leads to abnormalities in multiple organs in Klippel-Feil syndrome. As discussed above, the interplay between hyperactive GDF6 prodomain signaling and GDF6 BMP signaling may need to be considered when dissecting the pathogenesis of Klippel-Feil syndrome.

The results of this study also suggest that GDF6 is a potential therapeutic target for Ewing sarcoma. Although EWS-FLI1 functions as a driver of Ewing sarcoma, it is notoriously difficult to target and remains the “perfect target without a therapeutic agent” (Uren and Toretsky, 2005). One way to resolve this conundrum is to identify and target the dependency created by EWS-FLI1. GDF6 emerged as such dependency created by EWS-FLI1: *GDF6* is a direct transcriptional activation target of EWS-FLI1 and is highly expressed in Ewing sarcoma (Figure 1), and silencing of GDF6 profoundly impaired *in vitro* and *in vivo* growth of Ewing sarcoma (Figure 2). Being a secreted extracellular protein, GDF6 is likely amenable to therapeutic targeting. Indeed, anti-GDF6 prodomain antibody efficiently inhibited Ewing sarcoma growth (Figure 2B), suggesting the potential utility of GDF6 neutralizing antibodies as a therapeutic agent.

## STAR★METHODS

### RESOURCE AVAILABILITY

#### Lead Contact

Further information and requests for resources and reagents should be directed to and will be fulfilled by the Lead Contact, Yuzuru Shiio (shiio@uthscsa.edu).

#### Materials Availability

Plasmids generated in this study are available from the Lead Contact.

#### Data and Code Availability

This study did not generate datasets/code.

### EXPERIMENTAL MODEL AND SUBJECT DETAILS

#### Animals

Female 5 - 6 week old C.B.17SC scid−/− mice were used. All mice were housed in a pathogen-free vivarium in the University of Texas Health Science Center at San Antonio. Mice were randomly allocated to treatment groups. Blinding of the researcher measuring tumor size was employed. The animal research method was reviewed and approved for humaneness by the Institutional Animal Care and Use Committee of the University of Texas Health Science Center at San Antonio.

#### Cell Lines

A673, 293T, HeLa, HCT116, RD, U2OS, Saos-2, and Yamato cells were cultured in Dulbecco’s modified Eagle’s medium (DMEM) supplemented with 10% fetal bovine serum. EW8, TC32, TC71, SK-N-MC, CHLA-258, ES1, ES2, ES4, SK-PNET-Li, CHLA-352, and RH30 cells were cultured in RPMI-1640 medium supplemented with 10% fetal bovine serum. SK-ES1 and SK-NEP-1 cells were cultured in McCoy’s 5A modified medium supplemented with 15% fetal bovine serum. The cell lines were STR-authenticated and were routinely tested for the absence of mycoplasma. Cord blood-derived human mesenchymal stem cells were purchased from Vitro Biopharma (Golden, CO) and were cultured in low serum MSC-GRO following the manufacturer’s procedure.

### METHOD DETAILS

#### Transfection and viral infection

Calcium phosphate co-precipitation was used for transfection of 293T cells. Lentiviruses were prepared by transfection in 293T cells following System Biosciences’ protocol, and the cells infected with lentiviruses were selected with 2 μg/mL puromycin for 48 hours. cDNAsfor GDF6, GDF6 prodomain (residue 1 – 335 or 1 – 331), GDF6 BMP domain (residue 336 – 455, N-terminally fused to a signal peptide, residue 1 – 22), GDF5, GDF7, BMP2, BMP4, EWS-FLI1, CD99, CD99L2, CSK, and noggin were cloned into pcDNA3 mammalian expression vector (Invitrogen/Thermo Fisher) or pCDH1-puro lentiviral expression vector (System Biosciences). pCMV5 mouse src K295R Y527F was a gift from Joan Brugge and Peter Howley (Addgene plasmid # 13657; http://www.addgene.org/13657/; RRID:Addgene_13657). Src-Y530F was a gift from Cheng-han Yu (Addgene plasmid # 124659; http://www.addgene.org/124659/; RRID:Addgene_124659). pLP-EGFP-CSK was obtained from the DNA Resource Core at Harvard Medical School. The target sequences for shRNAs are as follows: FLI-1 C terminus shRNA, AACGATCAGTAAGAATACAGAGC; scrambled shRNA, CCTAAGGTTAAGTCGCCCTCG; GDF6 shRNA-1, GTGTCCATGCTCTCAGACAAA; and GDF6 shRNA-2, GCCAAGTGTTA CATTGAGCTT. The following siRNAs were used: human GDF6 siRNA SMARTpool (Cat# M-0330055-01-005; Dharmacon), human CSK siRNA SMARTpool (Cat# M-003110-02-0005; Dharmacon), human p21 siRNA SMARTpool (Cat# D-003471-00-0005, Dharmacon), and Non-Targeting siRNA Pool #2 (D-001206-14-05; Dharmacon). Lipofectamine RNAiMAX Transfection Reagent (Thermo Fisher) was used for siRNA transfection except for TC71 cells, which were transfected with TransIT®-siQUEST siRNA Transfection Reagent (Mirus Bio).

#### Protein sample preparation and proteomic analysis

The preparation of secreted protein samples, mass spectrometry analysis, and proteomics data processing were performed essentially as described (Elzi et al., 2016; Jayabal et al., 2017). Cells were washed six times with culture medium without serum. Subsequently, cells were cultured in medium without serum for 24 hours and the culture supernatant was harvested. The supernatant was centrifuged, filtered through a 0.45-μm filter (Millipore), and concentrated using 3,000-Da cut-off Amicon Ultra Centrifugal Filter Units (Millipore). The proteins in each sample were fractionated by SDS-PAGE and visualized by Coomassie blue. Each gel lane was divided into six slices, and the proteins in each slice were digested *in situ* with trypsin (Promega modified) in 40 mM NH_4_HCO_3_ overnight at 37°C. The resulting tryptic peptides were analyzed by HPLC-ESI-tandem mass spectrometry on a Thermo Fisher LTQ Orbitrap Velos Pro mass spectrometer. The Xcalibur raw files were converted to mzXML format and were searched against the UniProtKB/Swiss-Prot human protein database (UniProt release 2016_04) using X! TANDEM CYCLONE TPP (2011.12.01.1 - LabKey, Insilicos, ISB). Methionine oxidation was considered as a variable modification in all searches. Up to one missed tryptic cleavage was allowed. The X! Tandem search results were analyzed by the Trans-Proteomic Pipeline, version 4.3. Peptide/protein identifications were validated by the Peptide/ProteinProphet software tools (Keller et al., 2002; Nesvizhskii et al., 2003). A Protein-Prophet score of 0.9 was used as a cutoff, which corresponded to false discovery rates of 0.7%, 0.8%, 0.8%, 0.6%, 0.6%. 0.7%, and 0.7% in the A673, TC71, RH30, RD, U2OS, Saos-2, and Yamato datasets, respectively.

#### RNA samples and real-time quantitative RT-PCR

De-identified Ewing sarcoma tumor RNA samples were obtained from the Cooperative Human Tissue Network. Total cellular RNA was isolated using TRIzol reagent (Invitrogen). Reverse transcription was performed using a High Capacity cDNA Reverse Transcription Kit (Thermo Fisher) as per manufacturer’s instructions. Quantitative PCR was performed using PowerUp SYBR Green Master Mix (Thermo Fisher) on Applied Biosystems ViiA 7 Real-Time PCR System. Each sample was analyzed in triplicate. The following primers were used: *GDF6* forward 5′-CAGTCTTCCAAGTCGGCTAATAC-3′, *GDF6* reverse, 5′-CTGAGAGCATGGACACATCAA-3′; *EWS-FLI1* forward, 5′-GGCAGCAGAACCCTTCTTAT-3′, *EWS-FLI1* reverse, 5′-GGCCGTTGCTCTGTATTCTTA-3′; and *GAPDH* forward, 5′-GGTGTGAACCATGAGAAGTATGA-3′, *GAPDH* reverse, 5′-GAGTCCTTCCACGATACCAAAG-3′.

#### Immunoblotting and immunoprecipitation

Immunoblotting was performed as described (Jayabal et al., 2017). Immunoprecipitation was performed as described (Shiio and Eisenman, 2003). The following antibodies were used: rabbit anti-FLI-1 (ab15289, Abcam); rabbit anti-GDF6 (Novus, NBP 1-91934); rabbit anti-caspase 3 (9665, Cell Signaling Technology); rabbit anti-PARP (sc-7150, Santa Cruz Biotechnology); mouse anti-CD99 (MS-1633, Lab Vision); sheep anti-CD99L2 (AF5185, R&D Systems); mouse anti-p21 (BD PharMingen, 556430); mouse anti-tubulin (Developmental Studies Hybridoma Bank); rabbit anti-GAPDH (sc-25778, Santa Cruz Biotechnology); rabbit anti-CSK (HPA028425, Atlas Antibodies); mouse anti-GST (MS-707-P0, Lab Vision); mouse anti-GFP (sc-9996, Santa Cruz Biotechnology); rabbit anti-actin (8457, Cell Signaling Technology); rabbit anti-Src (2123, Cell Signaling Technology); rabbit anti-phospho-Src (2101, Cell Signaling Technology); rabbit anti-phospho-Cortactin (4569, Cell Signaling Technology); rabbit anti-phospho-p130 Cas (4011, Cell Signaling Technology); rabbit anti-phospho-STAT3 (9145, Cell Signaling Technology); rabbit anti-phospho-Smad1/5 (9516, Cell Signaling Technology); rabbit anti-HA (3724, Cell Signaling Technology); and mouse anti-FLAG (F1804, Sigma-Aldrich).

#### Immunofluorescence

Cells grown on coverslips were fixed with 4% paraformaldehyde for 15 minutes at room temperature, washed with PBS, and permeabilized and blocked with PBS containing 0.5% saponin and 5% normal goat serum for 45 minutes. The samples were incubated with the primary antibody (mouse anti-CD99 [MS-1633, Lab Vision] or mouse anti-FLAG [F1804, Sigma-Aldrich] as indicated) for one hour followed by goat anti-mouse IgG, DyLight™ 488 (35553, Thermo Scientific) for one hour. Nuclei were stained with DAPI. The images were collected with a FluoView FV3000 confocal laser scanning microscope (Olympus).

#### Chromatin-immunoprecipitation

Chromatin-immunoprecipitation (ChIP) was performed as described (Carey et al., 2009) using rabbit anti-FLI-1 antibody (ab15289, Abcam) or control rabbit IgG (ab37415, Abcam). The primer sequences used for ChIP are as follows: GDF6-#1 forward, 5′-GCATC GATCCAAACTCTAAAGAC-3′, *GDF6*-#1 reverse, 5′-GGTATCGCTCTCCACAAAGA-3′; *GDF6*-#2 forward, 5′-CTAAAGTCTGCGTCAGAAGAGG-3′, *GDF6*-#2 reverse, 5′-GGCCACGAGTCTTTAGAGTTT-3′; *NR0B1* forward, 5′-GTTTGTGCCTTCATGGGAAATGGTTATTC-3′, *NR0B1* reverse, 5′-CTAGTGTCTTGTGTGTCCCTAGGG-3′; *EHZ2* forward, 5′-GACACGTGCTTAGAACTACGAACAG-3′, *EZH2* reverse, 5′-TTTGGCTGGCCGAGCTT-3′; and *GAPDH* forward, 5′-GTGGTCTCCTCTGACTTCAAC-3′, *GAPDH* reverse, 5′-GTAGCCAAATTCGTTGTCATACC-3′.

#### Cell proliferation and xenograft tumorigenicity assays

Anchorage-dependent cell proliferation was assessed by IncuCyte live-cell imaging system (Essen BioScience). Anchorage-independent cell proliferation was evaluated by soft agar colony formation assays as described (Elzi et al., 2015). Sphere formation assays were done as described (Dasgupta et al., 2017) using ultra-low attachment 6-well plates (Corning; seeding density 1×10^4^ cells/well) and DMEM/F-12 medium supplemented with B27, human recombinant epidermal growth factor (20 ng/ml), and basic fibroblast growth factor (20 ng/ml). For xenograft tumorigenicity assays, cells were subcutaneously injected into the flanks of SCID mice (2 × 10^6^ cells/injection, five mice/group). Tumor growth was monitored weekly using a caliper. The animal research method was reviewed and approved for humaneness by the Institutional Animal Care and Use Committee.

#### Migration assays and Invasion assays

Migration assays were performed in modified Boyden chambers (Corning® Transwell®, 8 μm pore size, Sigma-Aldrich). Cells were seeded at 5 × 10^4^ cells/200 μL serum free-DMEM/F-12 medium in the upper chamber. The lower chamber was filled with DMEM/F-12 supplemented with 10% FBS. After 20-hour incubation, the membrane was gently removed from the chamber and the cells on the upper surface were removed using cotton swabs. Cells on the lower surface that migrated through the membrane were fixed with 50% methanol, stained with 0.1% crystal violet, and counted. For wound closure assays, a 96-pin WoundMaker (Essen Bioscience) was used to create homogeneous scratch wounds in a confluent monolayer of cells, and cell migration and wound closure were monitored by IncuCyte live-cell imaging system following Essen Bioscience’s protocol. Matrigel invasion assays were performed following Corning’s protocol.

#### Ligand binding assays

The GDF6 prodomain-alkaline phosphatase fusion protein or unfused alkaline phosphatase was produced by transfection in 293T cells. A673 cells were washed twice with AP binding buffer (20 mM HEPES (pH 7.0), 0.2% BSA, 5mM CaCl_2_, 1 mM MgCl_2_, and 2 μg/ml heparin) and incubated with the GDF6 prodomain-alkaline phosphatase fusion protein or unfused alkaline phosphatase in AP binding buffer for 90 minutes at 4°C. After three washes with AP binding buffer, cells were fixed with 4% paraformaldehyde in PBS for 15 minutes and washed three times with 20 mM HEPES (pH 7.0) and 150 mM NaCl. Endogenous alkaline phosphatase activity was inactivated by incubation at 65°C for 3 hours, cells were washed twice with AP buffer (100 mM Tris (pH 9.5), 100 mM NaCl, and 50 mM MgCl2), and alkaline phosphatase activity was visualized by incubating the cells overnight with NBT/BCIP (Roche; used at 1:50 dilution) in AP buffer. The reaction was stopped by three washes with 1 mM EDTA and 0.1% Triton X-100 in PBS.

#### Proximity ligation assays

Proximity ligation assays were performed using a Duolink *In Situ* Red Starter Mouse/Rabbit kit (Sigma-Aldrich) following the manufacturer’s instructions. Briefly, cells were plated on 8-well chamber slides and transfected with siRNAs or plasmids and/or treated with conditioned medium as indicated. Forty-eight hours after transfection, cells were fixed with 4% paraformaldehyde for 15 minutes, washed with PBS, permeabilized with PBS containing 0.5% saponin for 45 minutes, and washed twice with wash buffer A. Samples were incubated with blocking solution at 37°C for 30 minutes and washed twice with wash buffer A. Subsequently, samples were incubated with rabbit anti-HA antibody (3724, Cell Signaling Technology) and mouse anti-CD99 antibody (MS-1633, Lab Vision) or mouse anti-FLAG antibody (F1804, Sigma-Aldrich) diluted in Duolink antibody diluent for one hour at 37°C. After two washes with wash buffer A, samples were incubated with two PLA probes diluted in Duolink antibody diluent for one hour at 37°C. After two washes, samples were incubated with ligase diluted in ligation buffer for 30 minutes at 37°C followed by incubation with polymerase in amplification buffer for 100 minutes at 37°C. Finally, samples were washed twice with 1x wash buffer A, twice with 1xwash buffer B, and once with 0.1x wash buffer B. The images were collected with a FluoView FV3000 confocal laser scanning microscope (Olympus).

#### Microscale thermophoresis

Recombinant human CD99-Fc fusion protein (3968-CD-050, residue 23 – 122, R&D Systems) was labeled using the protein labeling kit (RED-NHS 2^nd^ generation, MO-L011, Nano Temper) according to the manufacturer’s protocol. The GDF6 prodomain (residue 1 – 335), GDF6 A249E prodomain, or GDF6 L289P prodomain was produced by transfection in 293T cells, serially diluted in Opti-MEM (31985-070, Life Technologies), and combined with labeled CD99-Fc fusion protein in PBS/0.005% Tween-20. After 30-minute incubation at room temperature, the samples were loaded to the capillary chips of the Monolith NT.115 instrument (NanoTemper). The data analysis was performed using MO Affinity Analysis Software (NanoTemper).

### QUANTIFICATION AND STATISTICAL ANALYSIS

Statistical analyses were performed using the exact Wilcoxon – Mann – Whitney test (Marx et al., 2016) except for the Kaplan – Meier analysis in Figure S5, which used the log-rank test. Data are expressed as mean ± SEM. The results were considered significant when p < 0.05. The number of replicates, independent samples, and animals is indicated in the figure legends.

## Supplementary Material

1

2

## Figures and Tables

**Table:** KEY RESOURCES TABLE

REAGENT or RESOURCE	SOURCE	IDENTIFIER
Antibodies		
anti-FLI-1	Abcam	ab15289
anti-GDF6	Novus	NBP 1-91934
anti-caspase 3	Cell Signaling Technology	9665
anti-PARP	Santa Cruz Biotechnology	sc-7150
anti-CD99	Lab Vision	MS-1633
anti-CD99L2	R&D Systems	AF5185
anti-p21	3D PharMingen	556430
anti-tubulin	Developmental Studies Hybridoma Bank	N/A
anti-GAPDH	Santa Cruz Biotechnology	sc-25778
anti-CSK	Atlas Antibodies	HPA028425
anti-GST	Lab Vision	MS-707-P0
anti-GFP	Santa Cruz Biotechnology	sc-9996
anti-actin	Cell Signaling Technology	8457
anti-Src	Cell Signaling Technology	2123
anti-phospho-Src	Cell Signaling Technology	2101
anti-phospho-Cortactin	Cell Signaling Technology	4569
anti-phospho-p130 Cas	Cell Signaling Technology	4011
anti-phospho-STAT3	Cell Signaling Technology	9145
anti-phospho-Smad1/5	Cell Signaling Technology	9516
anti-HA	Cell Signaling Technology	3724
anti-FLAG	Sigma-Aldrich	F1804
goat anti-mouse IgG, DyLight™ 488	Thermo Fisher Scientific	35553
control rabbit IgG	Abcam	ab37415
Bacterial and Virus Strains		
DH10B	Thermo Fisher Scientific	12331013
Biological Samples		
De-identified Ewing sarcoma tumor RNA samples	Cooperative Human Tissue Network	N/A
Chemicals, Peptides, and Recombinant Proteins		
puromycin dihydrochloride	Sigma-Aldrich	P8833
dasatinib	Apexbio Technology	BMS-354825
PD98059	Cell Signaling Technology	9900
Lipofectamine RNAiMAX Transfection Reagent	Thermo Fisher Scientific	13778150
TransIT®-siQUEST siRNA Transfection Reagent	Mirus Bio	MIR 2114
TRIzol Reagent	Invitrogen/Thermo Fisher Scientific	15596026
Critical Commercial Assays		
High Capacity cDNA Reverse Transcription Kit	Thermo Fisher Scientific	4368814
PowerUp SYBR Green Master Mix	Thermo Fisher Scientific	A25741
Duolink *In Situ* Red Starter Kit Mouse/Rabbit	Sigma-Aldrich	DUO92101
Monolith Protein Labeling Kit RED-NHS 2nd Generation (Amine Reactive)	NanoTemper	MO-L011
Experimental Models: Cell Lines		
A673	ATCC	CRL-1598
293T	ATCC	CRL-11268
HeLa	ATCC	CCL-2
EW8	Dr. Patrick Grohar	N/A
TC32	Dr. Patrick Grohar	N/A
TC71	Coriell Institute	GM11654
SK-N-MC	ATCC	HTB-10
CHLA-258	Childhood Cancer Repository	N/A
ES1	Dr. Peter Houghton	N/A
ES2	Dr. Peter Houghton	N/A
ES4	Dr. Peter Houghton	N/A
SK-ES-1	ATCC	HTB-86
HCT116	ATCC	CCL-247
SK-PNET-Li	Dr. Javed Khan	N/A
CHLA-352	Dr. Javed Khan	N/A
RH30	ATCC	CRL-2061
RD	ATCC	CCL-136
U2OS	ATCC	HTB-96
Saos-2	ATCC	HTB-85
Yamato-SS	RIKEN Bioresource Center	RCB3577
SK-NEP-1	ATCC	HTB-48
Cord blood-derived human mesenchymal stem cells	Vitro Biopharma	SC00A1
Experimental Models: Organisms/Strains		
C.B.17SC scid ^−/−^ mice, 5-6 weeks old	Taconic Biosciences	CB17SC
Oligonucleotides		
*GDF6* forward, CAGTCTTCCAAGTCGGCTAATAC	Thermo Fisher Scientific	N/A
*GDF6* reverse, CTGAGAGCATGGACACATCAA	Thermo Fisher Scientific	N/A
*EWS-FLI1* forward, GGCAGCAGAACCCTTCTTAT	Thermo Fisher Scientific	N/A
*EWS-FLI1* reverse, GGCCGTTGCTCTGTATTCTTA	Thermo Fisher Scientific	N/A
*GAPDH forward*, GGTGTGAACCATGAGAAGTATGA	Thermo Fisher Scientific	N/A
*GAPDH* reverse, GAGTCCTTCCACGATACCAAAG	Thermo Fisher Scientific	N/A
*GDF6*-#1 ChIP forward, GCATCGATCCAAACTCTAAAGAC	Thermo Fisher Scientific	N/A
*GDF6*-#1 ChIP reverse, GGTATCGCTCTCCACAAAGA	Thermo Fisher Scientific	N/A
*GDF6*-#2 ChIP forward, CTAAAGTCTGCGTCAGAAGAGG	Thermo Fisher Scientific	N/A
*GDF6*-#2 ChIP reverse, GGCCACGAGTCTTTAGAGTTT	Thermo Fisher Scientific	N/A
*NR0B1* ChIP forward, GTTTGTGCCTTCATGGGAAATGGTTATTC	Thermo Fisher Scientific	N/A
*NR0B1* ChIP reverse, CTAGTGTCTTGTGTGTCCCTAGGG	Thermo Fisher Scientific	N/A
*EHZ2* ChIP forward, GACACGTGCTTAGAACTACGAACAG	Thermo Fisher Scientific	N/A
*EZH2* ChIP reverse, TTTGGCTGGCCGAGCTT	Thermo Fisher Scientific	N/A
*GAPDH* ChIP forward, GTGGTCTCCTCTGACTTCAAC	Thermo Fisher Scientific	N/A
*GAPDH* ChIP reverse, GTAGCCAAATTCGTTGTCATACC	Thermo Fisher Scientific	N/A
Recombinant DNA		
pcDNA3.1 vector	Invitrogen/Thermo Fisher Scientific	V79020
pCDH1-MCS1-EF1-Puro vector	System Biosciences	CD510A-1
pCMV5 mouse src K295R Y527F	Addgene	13657
Src-Y530F	Addgene	124659
pLP-EGFP-CSK	The DNA Resource Core at Harvard Medical School	HsCD00035985

## Highlights

- The autocrine signaling mediated by GDF6 maintains Ewing sarcoma growth
- The GDF6 prodomain is a ligand for CD99, a widely used marker for Ewing sarcoma
- GDF6 triggers CSK recruitment to CD99 intracellular domain, inhibiting Src activity
- Klippel-Feil syndrome-linked GDF6 mutants are hyperactive in CD99-Src signaling
