# Supplementary material for: GDF6-CD99 Signaling Regulates Src and Ewing Sarcoma Growth
Source: Cell Rep. Author manuscript; Available in PMC 2020 Nov 25. (PMC7688343; doi:10.1016/j.celrep.2020.108332)
Supplement: 1 [file NIHMS1643870-supplement-1.pdf]

Cell Reports, Volume *33*

Supplemental Information

GDF6-CD99 Signaling Regulates Src and Ewing Sarcoma Growth

Fuchun Zhou, David J. Elzi, Panneerselvam Jayabal, Xiuye Ma, Yu-Chiao Chiu, Yidong Chen, Barron Blackman, Susan T. Weintraub, Peter J. Houghton, and Yuzuru Shiio

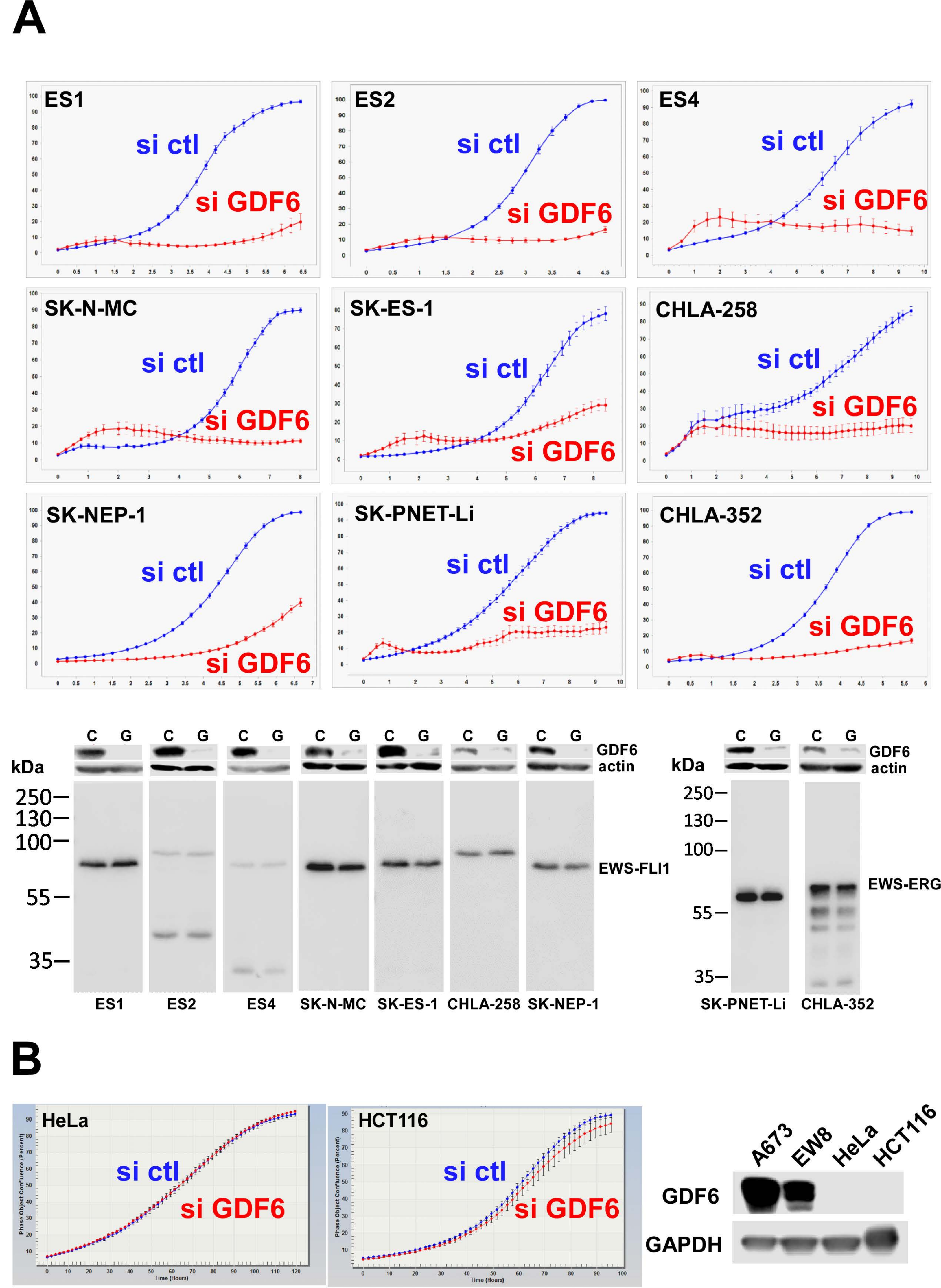
A
ES1
si ctl
si GDF6
ES2
si ctl
si GDF6
ES4
si ctl
si GDF6
SK-N-MC
si ctl
si GDF6
SK-ES-1
si ctl
si GDF6
CHLA-258
si ctl
si GDF6
SK-NEP-1
si ctl
si GDF6
SK-PNET-Li
si ctl
si GDF6
CHLA-352
si ctl
si GDF6
C G
GDF6
actin
kDa
250
130
100
55
35
EWS-FLI1
ES1
ES2
ES4
SK-N-MC
SK-ES-1
CHLA-258
SK-NEP-1
EWS-ERG
SK-PNET-Li
CHLA-352
B
HeLa
si ctl
si GDF6
Phase Object Confluence (Percent)
Time (Hours)
HCT116
si ctl
si GDF6
A673
EW8
HeLa
HCT116
GDF6
GAPDH

Figures S1 GDF6 silencing inhibits Ewing sarcoma growth, related to Figure 2.

A. Indicated Ewing sarcoma cells were transfected with GDF6 siRNA or control siRNA and cell proliferation was assessed by IncuCyte (top). GDF6 silencing and the expression of EWS-FLI1 or EWS-ERG were verified by immunoblotting (bottom). C: control siRNA; G: GDF6 siRNA

B. GDF6 siRNA does not affect the proliferation of HeLa and HCT 116 cells (left), which do not express GDF6 protein (right).

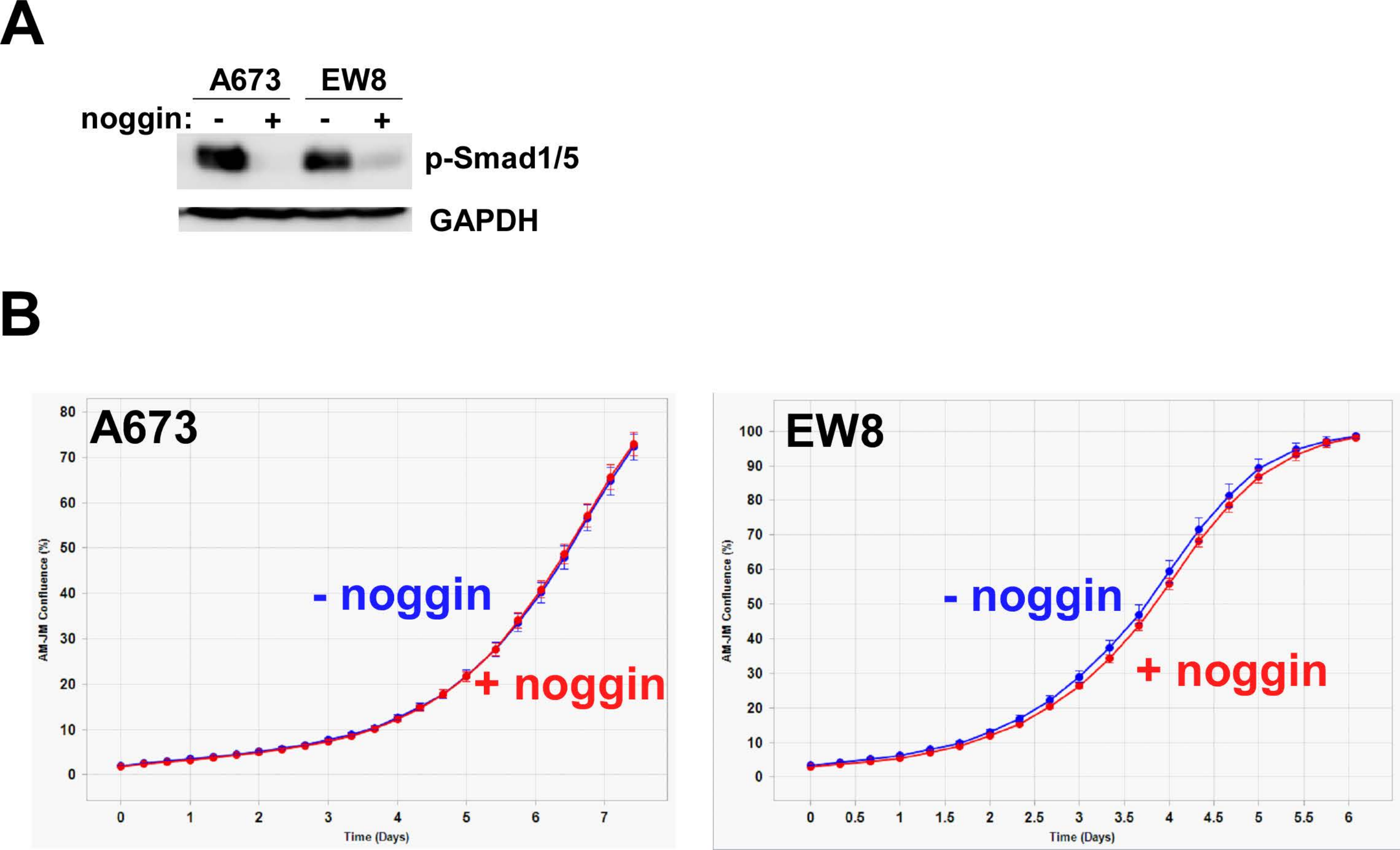
A
A673
EW8
noggin: - + - +
p-Smad1/5
GAPDH
B
A673
- noggin
+ noggin
AM-JM Confluence (%)
Time (Days)
EW8
- noggin
+ noggin
AM-JM Confluence (%)
Time (Days)

Figure S2 Ewing sarcoma does not depend on BMP signaling, related to Figure 2.

A. Noggin abrogates phosphorylated Smad1/5 in A673 and EW8 cells. A673 and EW8 cells were treated with 293T cell conditioned medium expressing noggin or vector and the levels of phopho-Smad1/5 were assessed by immunoblotting.

B. Noggin does not affect A673 and EW8 cell proliferation. A673 and EW8 cells were treated with noggin or control conditioned medium as in A and cell proliferation was assessed by IncuCyte.

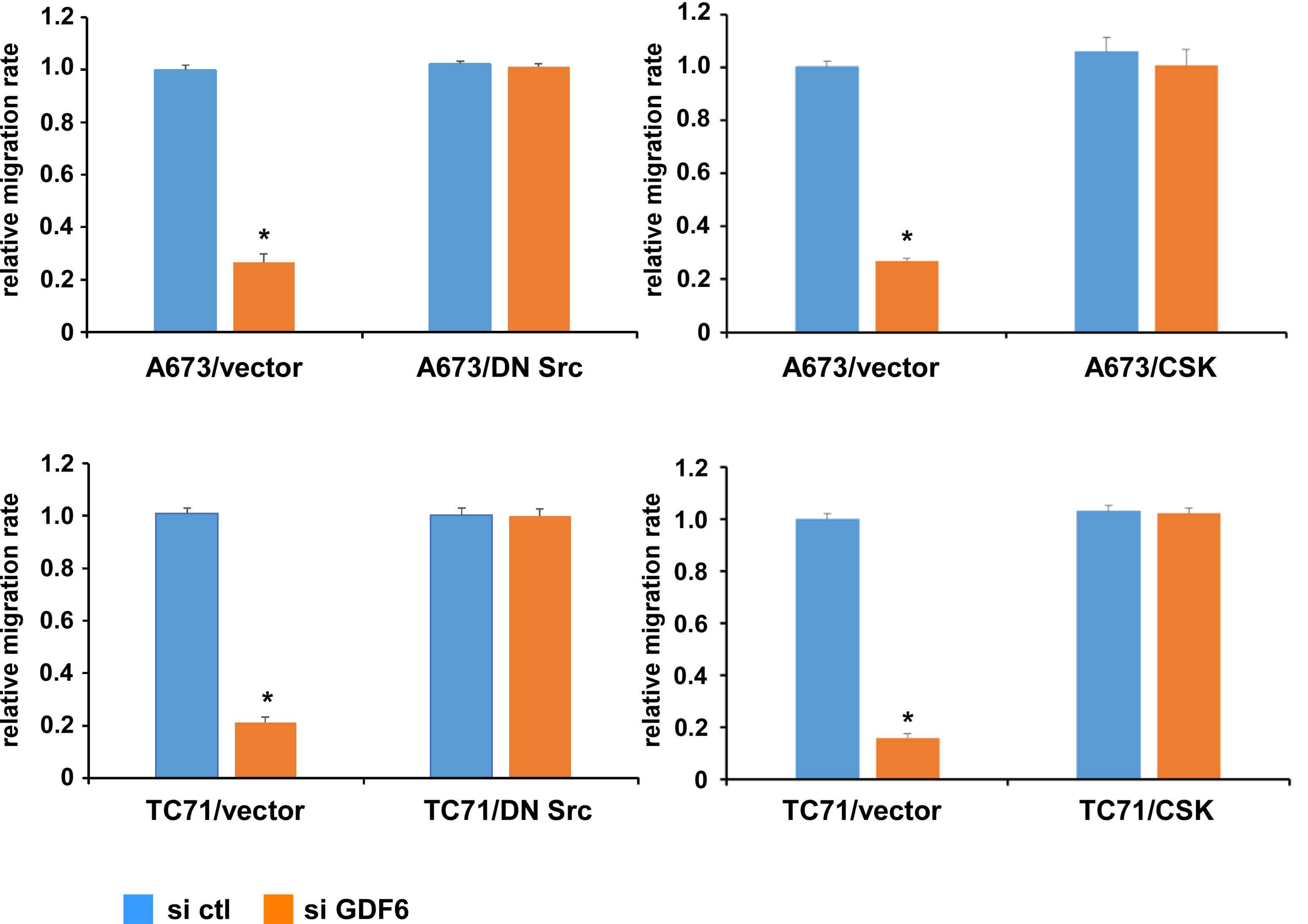
relative migration rate
1.2
1.0
0.8
0.6
0.4
0.2
0
*
A673/vector
A673/DN Src
A673/CSK
TC71/vector
TC71/DN Src
TC71/CSK
si ctl
si GDF6

Figure S3 Inhibition of cell migration by GDF6 silencing was abolished by dominant negative Src and by CSK, related to Figures 2 and 4.

A673 and TC71 cells stably expressing dominant negative Src, CSK, or empty vector were transfected with GDF6 siRNA or control siRNA, and cell migration was assessed by Transwell assays. * $p < 0.05$ ($n = 5$)

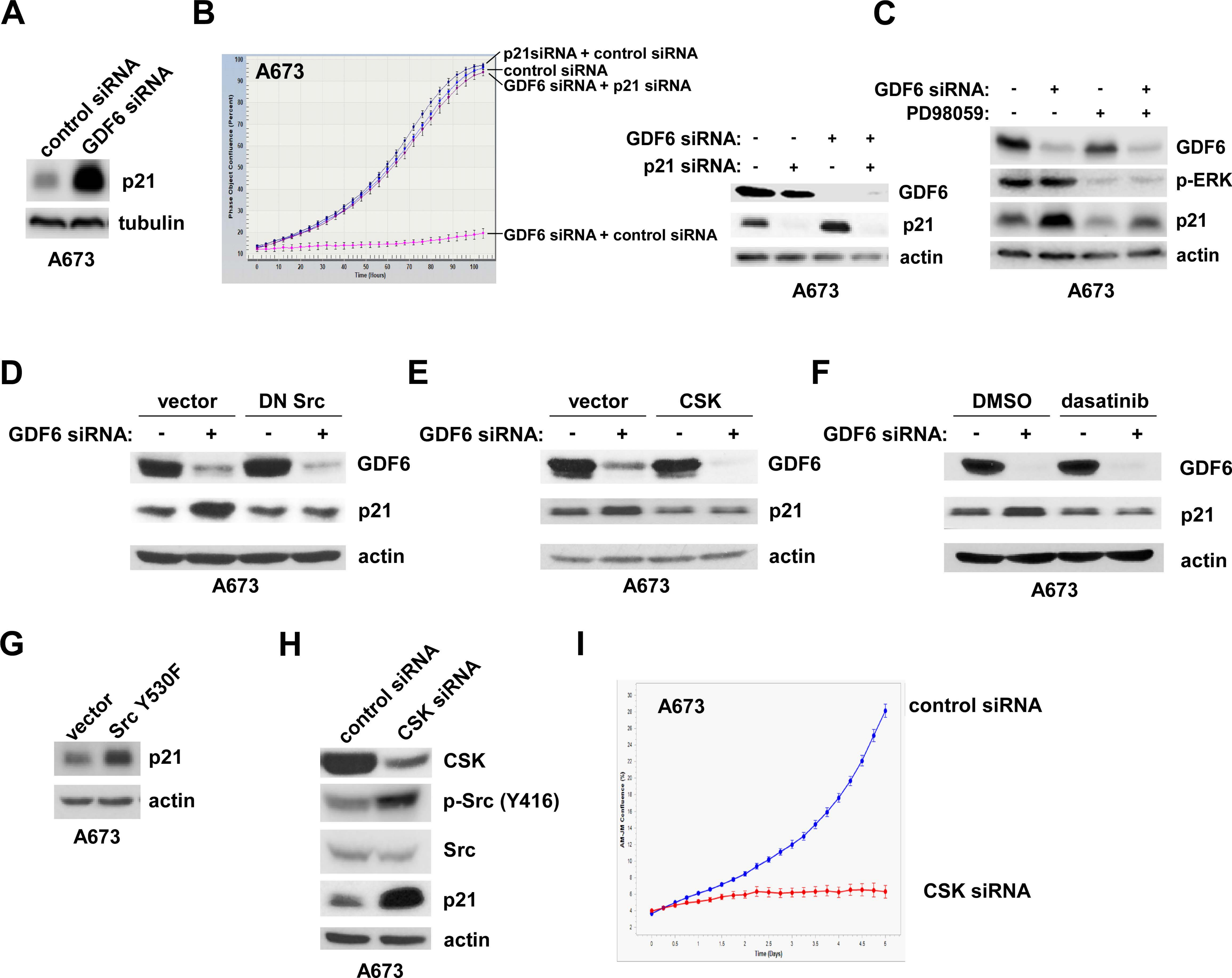

A
control siRNA
GDF6 siRNA
p21
tubulin
A673
B
A673
p21siRNA + control siRNA
control siRNA
GDF6 siRNA + p21 siRNA
GDF6 siRNA + control siRNA
Phase Object Confluence (Percent)
Time (Hours)
GDF6 siRNA: - - + +
p21 siRNA: - + - +
GDF6
p21
actin
A673
C
GDF6 siRNA: - + - +
PD98059: - - + +
GDF6
p-ERK
p21
actin
A673
D
vector
DN Src
GDF6 siRNA: - + - +
GDF6
p21
actin
A673
E
vector
CSK
GDF6 siRNA: - + - +
GDF6
p21
actin
A673
F
DMSO
dasatinib
GDF6 siRNA: - + - +
GDF6
p21
actin
A673
G
vector
Src Y530F
p21
actin
A673
H
control siRNA
CSK siRNA
CSK
p-Src (Y416)
Src
p21
actin
A673
I
A673
control siRNA
CSK siRNA
AM-JM Confluence (%)
Time (Days)

Figure S4 GDF6 silencing results in p21-dependent growth arrest of Ewing sarcoma through Src hyperactivation, related to Figure 4.

A. GDF6 silencing induces p21 in A673 cells.
B. Growth arrest induced by GDF6 silencing is abolished by p21 silencing. A673 cells were transfected with GDF6 siRNA, p21 siRNA, or control siRNA as indicated, and cell proliferation was assessed by IncuCyte (left). The silencing of GDF6 and p21 was verified by immunoblotting (right).
C. GDF6 silencing induces p21 upon MAP kinase pathway blockade by a MEK inhibitor, PD98059. PD98059 was used at 5 μM. The blockade of MAP kinase pathway was verified by suppression of phosphorylated ERK.
D. Dominant negative Src abrogates p21 induction by GDF6 silencing in A673 cells.
E. CSK abrogates p21 induction by GDF6 silencing in A673 cells.
F. Dasatinib abrogates p21 induction by GDF6 silencing in A673 cells. Dasatinib was used at 50 nM.
G. Src Y530F induces p21 in A673 cells.
H. CSK silencing activates Src and induces p21 in A673 cells. A673 cells were transfected with CSK siRNA or control siRNA and the levels of indicated proteins were assessed by immunoblotting.
I. CSK silencing inhibits A673 cell proliferation. A673 cells were transfected with CSK siRNA or control siRNA and cell proliferation was assessed by IncuCyte.

Overall survival (OS)

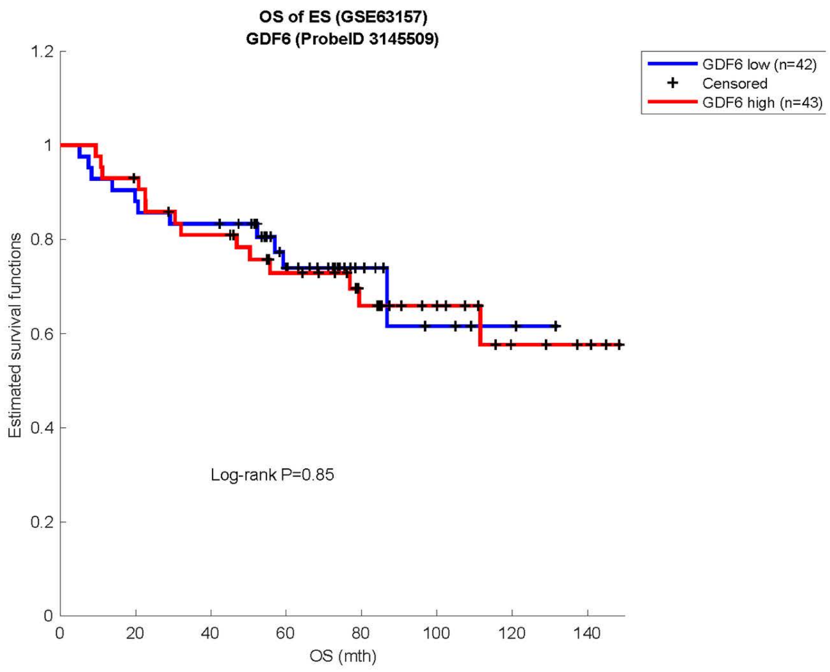

Event-free survival (EFS)

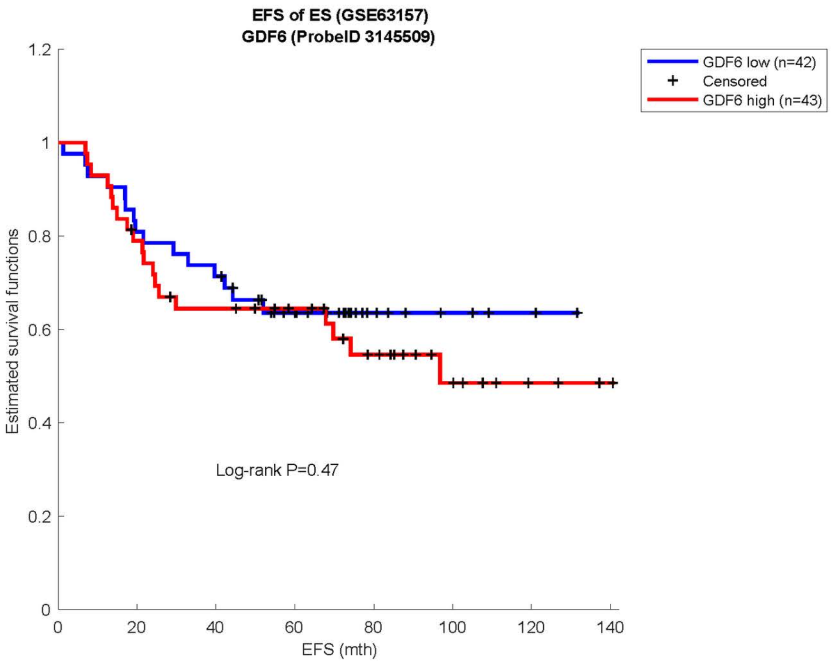

Fig. S5 GDF6 expression levels in Ewing sarcoma tumors and patient survival, related to Figure 1.

The Kaplan – Meier analysis of GDF6 expression levels in Ewing sarcoma tumors and patient survival. (n = 85, accession ID: GSE63157)
